# Exciplex-Forming Systems of Physically Mixed and Covalently Bonded Benzoyl-1*H*-1,2,3-Triazole and Carbazole Moieties for Solution-Processed White OLEDs

**DOI:** 10.1021/acs.joc.1c02784

**Published:** 2022-03-04

**Authors:** Mariia Stanitska, Malek Mahmoudi, Nazariy Pokhodylo, Roman Lytvyn, Dmytro Volyniuk, Ausra Tomkeviciene, Rasa Keruckiene, Mykola Obushak, Juozas Vidas Grazulevicius

**Affiliations:** † Department of Polymer Chemistry and Technology, Kaunas University of Technology, Baršausko Str. 59, LT-51423 Kaunas, Lithuania; ‡ Ivan Franko National University of Lviv, Kyryla i Mefodiya 6, 79005 Lviv, Ukraine

## Abstract

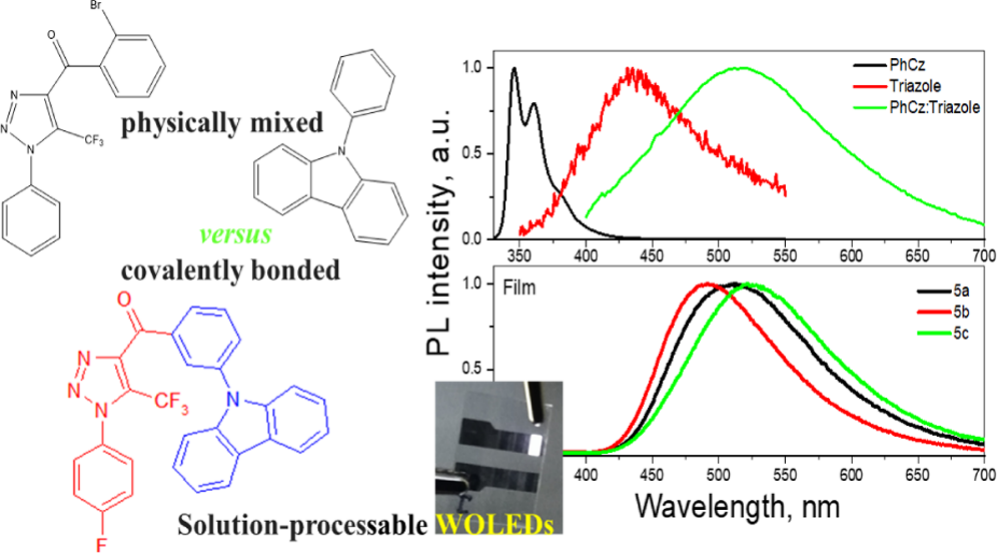

Using the newly designed exciplex-forming 1,2,3-triazole-based acceptors with fast and efficient singlet → triplet intersystem crossing (ISC) processes, carbazole and benzoyl-1*H*-1,2,3-triazole derivatives were synthesized by Dimroth-type 1,2,3-triazole ring formation and Ullmann–Goldberg C–N coupling reactions. Due to the exciplex formation between covalently bonded electron-donating (carbazole) and 1,2,3-triazole-based electron-accepting moieties with small singlet-triplet splitting (0.07–0.13 eV), the compounds exhibited ISC-assisted bluish–green thermally activated delayed fluorescence. The compounds were characterized by high triplet energy levels ranging from 2.93 to 2.98 eV. The most efficient exciplex-type thermally activated delayed fluorescence was observed for ortho-substituted carbazole-benzoyl-1*H*-1,2,3-triazole which was selected as a host in the structure of efficient solution-processed white light-emitting diodes. The best device exhibited a maximum power efficiency of 10.7 lm/W, current efficiency of 18.4 cd/A, and quantum efficiency of 7.1%. This device also showed the highest brightness exceeding 10 thousand cd/m^2^. Usage of the exciplex-forming host allowed us to achieve a low turn-on voltage of 3.6 V. High-quality white electroluminescence was obtained with the close to nature white color coordinates (0.31, 0.34) and a color rendering index of 92.

## 1 Introduction

White organic light emitting diodes (WOLEDs) are promising candidates for the next-generation solid-state lighting devices. They have attracted great attention from academia and industry thanks to their promising characteristics, such as low power consumption, flexibility, light weight, and high color quality.^1−3^ Recently, WOLEDs have entered the mainstream display market, since they can show comparable performance with the liquid crystal displays and GaN-based LEDs.^4−7^ An appealing feature of WOLED technology is the solution processibility of components. The solution-processed OLEDs have the potential to be printed into complex structures and shapes of light-emitting areas/pixels^8^ taking into account that single-layer devices can be fabricated by solution processing; there has been a large amount of work in this research area, using blends^9,10^ or co-polymers^11,12^ for emissive layers. Meanwhile, these approaches suffer from strong voltage-dependent color shifts.^13,14^ In contrast, in multilayer devices where one or more evaporated layers are combined with one or two solution-processed layers (hybrid devices), higher efficiencies and better color stability (but still not sufficient) are obtainable.^15,16^ Much efforts were devoted to the development and performance of hybrid WOLEDs by modifying their structures and optimizing layers such as hole-transporting, hole-blocking, electron-transporting, to achieve an effective and balanced carrier injection.^17,18^ In addition to electroluminescent (EL) efficiencies and lifetime, the color of emission is another significant aspect of OLED performance. For high-quality white-light illumination, sources with the International Commission on Illumination commonly abbreviated as CIE coordinates similar to that of blackbody radiation with a correlated color temperature (CCT) between 2500 and 6500 K, and a CRI above 80 is required.^19−22^ The color rendering index (CRI) scale ranges from 0 to 100 and describes the ability of the light source to exhibit colors realistically in comparison with a standard incandescent lamp.^23,24^ It is also a known fact that to realize high CRI, a WOLED should have an as broad as possible emission spectrum.^25,26^ Ultra-high CRI is important for lighting applications in museums, art galleries, and other commercial places. Great developments in efficient white OLEDs with a CRI value higher than 90 were recently observed.^27,28^

Recent progress in the design and synthesis of materials for OLEDs is closely connected with exploration of new donor–acceptor (D–A) systems.^29^ The effect of intramolecular charge transfer in excited states which is the result of D–A architecture has been widely used in the design of the compounds with numerous practically valuable photophysical properties including thermally activated delayed fluorescence (TADF),^30,31^ room-temperature phosphorescence,^32^ hybridized local and charge-transfer (HLCT),^33^ and twisted intramolecular charge transfer.^34^ In the design of TADF compounds, triazines,^35^ cyanobenzenes,^36^ benzophenones,^37^ diphenylsulphones,^38^ and other moieties^39^ were used as acceptor units.

When a 6-cyano-9-phenylpurine (PCP) acceptor unit was linked to a carbazole-based donor unit, the obtained exciplex-forming compounds with covalently bonded donor and acceptor moieties demonstrated sub-microsecond TADF with delayed-only emission and high reverse intersystem crossing (RISC) rates exceeding 10^7^ s^–1^.^40^ Such fast and efficient intersystem crossing (ISC) was achieved due to highly efficient population of a local excited triplet state (^3^LE_A_) of PCP from the singlet state ^1^LE_A_ of the acceptor (PCP). The locally excited triplet state ^3^LE_A_ provides an efficient RISC pathway (^3^LE_A_ → ^1^CT) between the excited singlet intermolecular charge transfer state (^1^CT) of the PCP-based exciplex systems. These exciplex-forming systems are characterized by the reduced lifetime of TADF similar to that of prompt fluorescence as well as improved color purity as for exciplex-based or conventional TADF. However, relatively strong TADF quenching was observed for the PCP-based exciplex systems limiting their photoluminescence (PL) quantum yields due to the limited triplet state stability of PCP at room temperature. We predict that such limitations can be overcome if acceptors with more stable triplet states are developed.

With the proposal to verify the above prediction, new exciplex-forming 1,2,3-triazole-based acceptors were designed. These acceptors demonstrated very weak fluorescence because of the fast and ISC. In addition, their local excited triplet states ^3^LE_A_ were practically equal in energy to the intermolecular charge transfer state ^1^CT of exciplex systems (solid molecular mixture of the triazole acceptor and phenylcarbazole donor) allowing an efficient RISC pathway ^3^LE_A_ → ^1^ICT of exciplex-forming systems with covalently bonded donor and acceptor moieties. The exciplex-forming properties were investigated for both physically mixed donor and acceptor and for the compounds covalently bonded systems with a carbazole-based donor and new triazole-based acceptors. The synthesis, electrochemical, thermal, and photophysical properties of a series of derivatives of benzoyl-1*H*-1,2,3-triazole and carbazole are reported. To the best of our knowledge, the benzoyl-1*H*-1,2,3-triazole moiety was not yet used as acceptor units in the design of TADF materials. 1,2,3-Triazole and its derivatives are characterized by high triplet energy values,^41^ which makes possible to use them for the development of not only emitters with the TADF effect but also of host materials for OLEDs. The most efficient exciplex-type TADF was observed for the derivative of ortho-substituted carbazole and benzoyl-1*H*-1,2,3-triazole which was selected as a host in the efficient solution-processed white light-emitting diodes. The hybrid WOLEDs with higher CRI than 90 was developed by the careful adjustment of the concentration ratio of host and light-emitting components in the emitting layer.

## 2 Results and Discussion

### 2.1 Synthesis

**Figure 1:**
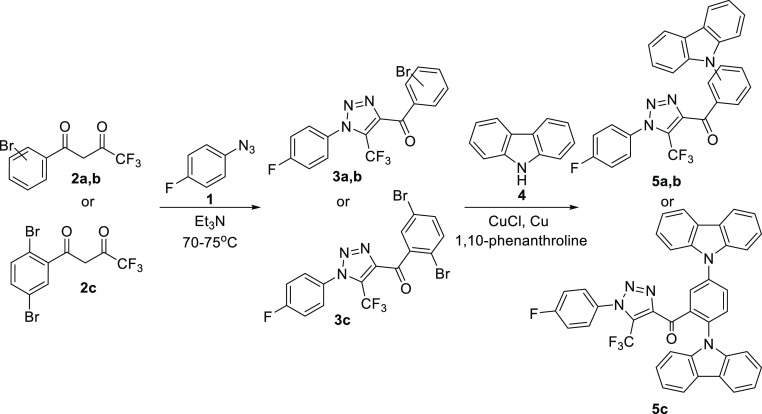
General synthetic scheme of benzoyl-1*H*-1,2,3-triazole and carbazole derivatives.

Synthesis of the target derivatives of benzoyl-1*H*-1,2,3-triazole and carbazole was based on convenient Dimroth-type 1,2,3-triazole synthesis^42^ and the following Ullmann–Goldberg C–N coupling (Figure 1).^43^ Based on recent works,^44,45^ 4-fluorophenyl azide **1** was chosen as the azide component for the triazole formation. Appropriate reagents for the incorporation of the aryl moiety at position 4 of 1,2,3-triazole are 1,3-diketones. However, it is known that in the case of asymmetric 1,3-diketones, the mixture of isomeric 1,2,3-triazoles can be formed.^46^ Rosin et al. showed that usage of strong electron-withdrawing groups such as CF_3_ allows us to control the reaction direction and to obtain single 1,2,3-triazole.^47^ Another side reaction for Dimroth-type 1,2,3-triazole synthesis is the Regitz diazo transfer reaction, which can be avoided by choosing a base/solvent system, for example, mild organic bases such as trialkylamines or alkali metal carbonates.^48^ Based on the previous experience, two systems K_2_CO_3_/DMSO and the triethylamine^47^ were selected for the reaction. The starting 1,3-diketones **2a**, **2b**, and **2c**, previously used for pyrazoline ring formation,^49^ were obtained from the corresponding bromine-substituted acetophenones by Claisen condensation with ethyl trifluoroacetate in almost quantitative yields.^50^ The reaction of 4-fluorophenyl azide **1** with 1-(3-bromophenyl)-4,4,4-trifluorobutane-1,3-dione **2b** yielded in 1,2,3-triazole **3b**. However, in triethylamine solution, the reaction was faster and the target triazole was isolated pure from the reaction mixture by simple filtration. Under these conditions, derivatives of ortho-substituted and dibromo-substituted 1,2,3-triazole (**3 a**,**c**) were obtained. Treatment of bromine-containing triazole derivatives **3** in Ullmann–Goldberg reaction with carbazole **4** yielded the target compounds **5a–c**. It should be noted that the reaction occurred selectively, and nucleophilic substitution of fluorine in the aryl substituent of triazole was not observed. The structures of the obtained triazoles **5a**-**c** were confirmed by ^1^H, ^13^C, and ^19^F NMR spectrometry. The data can be found in Supporting Information.

### 2.2 Theoretical Calculations and Electrochemical Properties

Before carrying out the syntheses of the designed structures, we estimated their properties by quantum chemical calculations. The density function theory calculations were performed using Gaussian’16 software. Geometries of the molecular structures were optimized at the B3LYP functional level with the 6–31G** basis set in vacuum.

**Figure 2:**
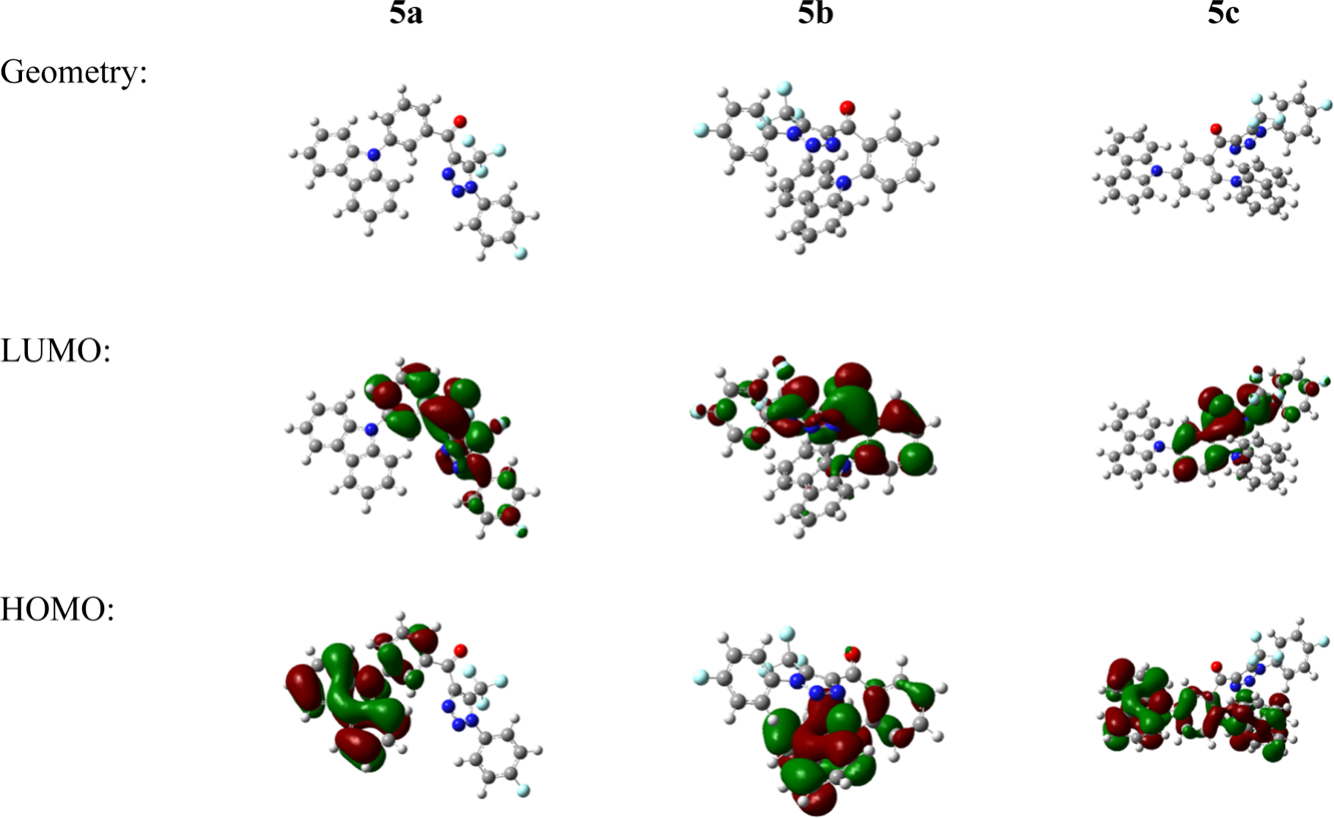
Distributions of HOMO and LUMO orbitals obtained by theoretical calculations.

The highest occupied molecular orbitals (HOMOs) and the lowest unoccupied molecular orbitals (LUMOs) were found to be slightly overlapped (Figure 2). The HOMOs were found to be located on electron-rich carbazole moieties and nearby phenyl ring of compounds **5a**, **5b**, and **5c**. The LUMOs were found to be delocalized on acceptor part that consists of electron-deficient triazole, carbonyl fragments, and 4-fluorophenyl rings. The energy levels of LUMOs were found to be close. The HOMO values were also found to be similar (Table 1). The calculated HOMO–LUMO gaps (3.12–3.24 eV) of the compounds favor their electron-injection and electron-transporting ability.

**Table 1:** Electrochemical Characteristics of Benzoyl-1*H*-1,2,3-Triazole and Carbazole Derivatives

	*E*_ox_, V	*E*_red_, V	IP, eV	EA, eV	*E*_G_^CV^, eV	HOMO, eV	LUMO, eV	Δ_|HOMO–LUMO|_, eV
**5a**	0.88	–1.62	5.98	3.09	2.87	–5.40	–2.27	3.12
**5b**	0.80	–1.53	5.90	3.16	2.56	–5.49	–2.05	3.44
**5c**	1.00	–2.21	6.10	2.89	3.21	–5.43	–2.18	3.24

**Figure 3:**
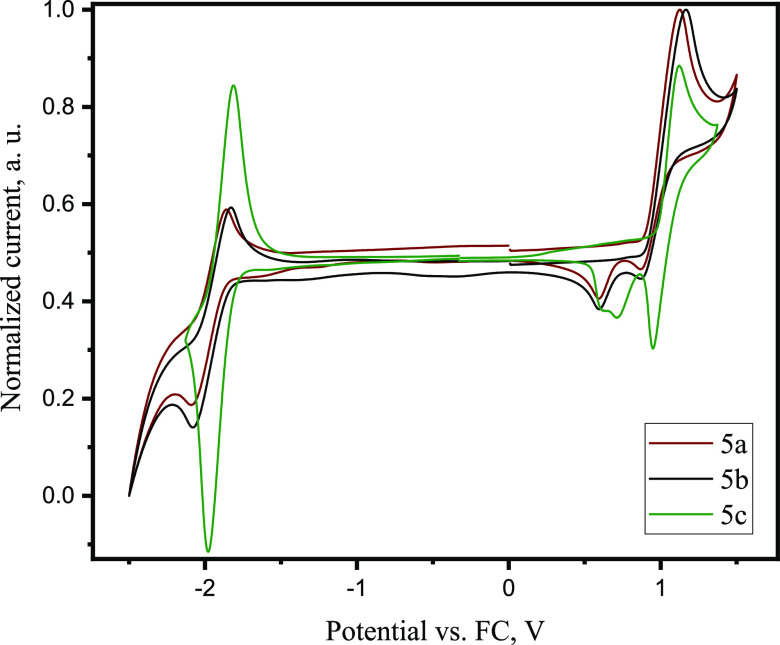
CV curves of compounds **5a**–**5c**.

To estimate electrochemical properties of the compounds, cyclic voltammetry (CV) measurements were carried out (Figure 3). It was established that the oxidation peak is approximately at the same position for all three compounds (**5a**, **5b**, and **5c**) and corresponds to the formation of radical cations of the carbazole moiety.

The differences of the IP values can be attributed to the different electron density distribution of the disubstituted triazole-based compound **5c** compared to those of mono-substituted triazole compounds **5a** and **5b**. The lowest ionization potential was observed for the o-isomer **5b** (Table 2), which can be explained by its specific configuration. The electron-donating carbazole moiety and electron-withdrawing carbonyl group are in ortho-position. This resulted in the increase of electron density on the carbazole fragment (in comparison to that of the m-isomer) and in the decrease of the ionization potential.

**Table 2:** Photophysical Characteristics of Benzoyl-1*H*-1,2,3-Triazole and Carbazole Derivatives^a^

	toluene/THF/films	toluene/films	compound (10 wt %):ZEONEX			films		
	λ_PL_, nm	PLQY, %	*E*_S1_, eV	*E*_T1_, eV	Δ*E*_ST_, eV	*E*_S1_, eV	*E*_T1_, eV	Δ*E*_ST_, eV
**5a**	480/507/510	11/12	2.61	2.6	0.01	2.82	2.76	0.06
**5b**	480/515/489	31/34	2.47	2.45	0.02	2.83	2.73	0.1
**5c**	482/343, 359, 378, 510/524	9/7	2.5	2.49	0.01	2.76	2.65	0.11

a λ_PL_, is the wavelength of the fluorescence intensity maxima; PLQY is the PL quantum yield; *E*_S1_ and *E*_T1_ are the energies of the first excited singlet and triplet states; and Δ*E*_ST_ is the singlet-triplet energy splitting.

The synthesized compounds (**5a**, **5b**, and **5c**) showed reversible reduction during CV scans. The values of electron affinity of the compounds were found to be close as they were determined by the presence of the triazole fragment. A smaller energy gap observed for **5c** relative to those estimated for **5a** and **5b** can probably be attributed to the increased overlap of frontier molecular orbitals (Figure 2) and subsequently stronger conjugation of chromophores in **5c**.

### 2.3 Photophysical Properties

**Figure 4:**
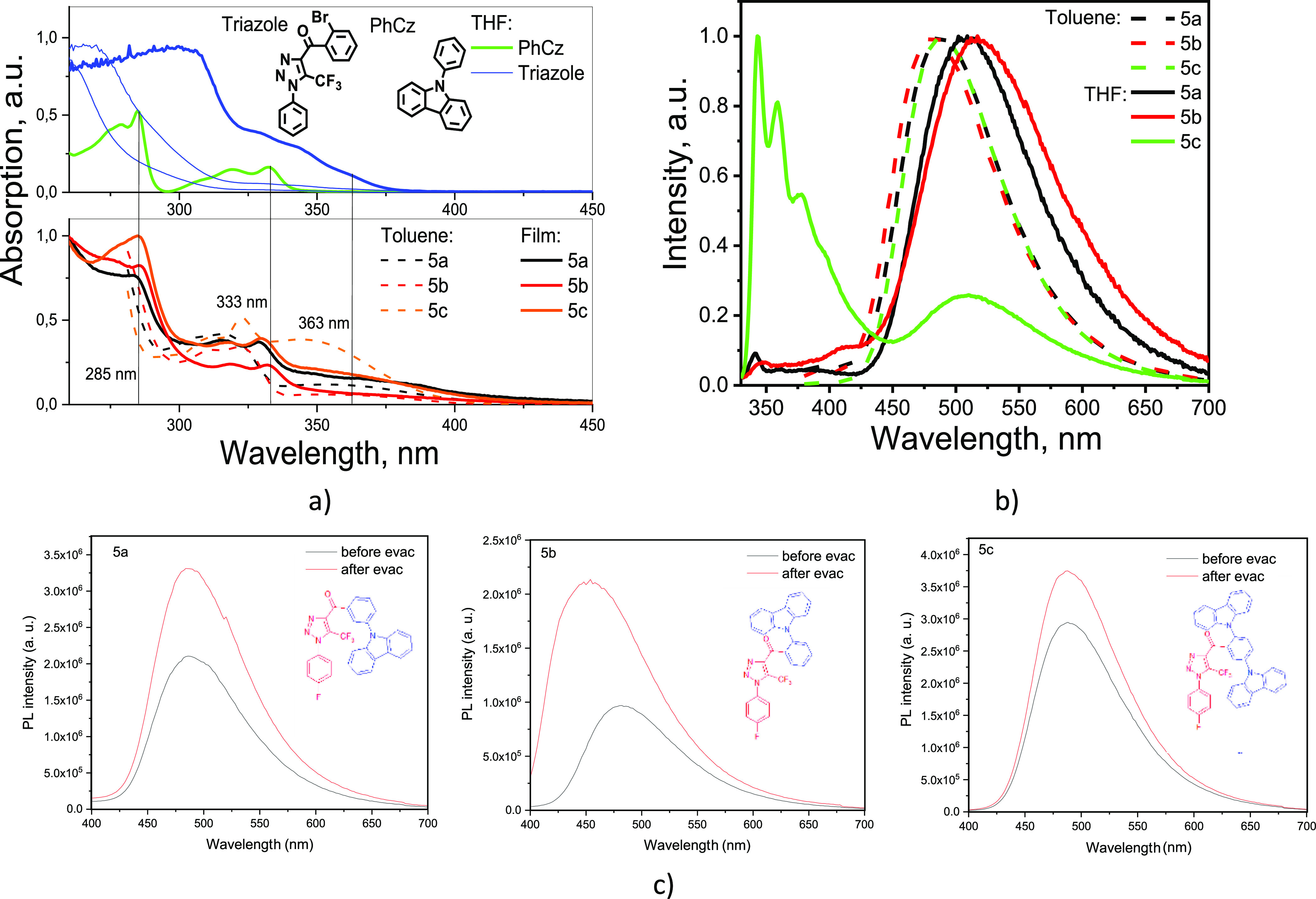
UV–vis (a) and PL (b) spectra of solid films and dilute solutions of **5a**–**5c**. PL spectra (c) of air equilibrated and deoxygenated toluene solutions of **5a**–**5c**. Excitation wavelengths of 350 nm (for toluene solutions) and 310 nm (for THF solutions).

Investigations of photophysical properties of **5a**–**5c** were started from recording of absorption and PL spectra of their dilute toluene solutions and solid films (Figure 4, Table 3). The toluene solutions of compounds **5a**, **5b**, and **5c** were found to absorb UV/Vis radiation up to 400 nm. Structured absorption spectra of the compounds showed maxima at 285 and 333 nm, which can mainly be assigned to π–π* and *n*–π* transitions of carbazole fragments, respectively (Figure 4a).^51^ This conclusion is well supported by the observation of the similar bands in absorption spectrum of the phenyl-carbazole (PhCz) moiety (Figure 4a). At first glance, the lowest energy bands (LEBs) with maxima near 350 nm for **5a**–**5c** could be attributed to intramolecular charge-transfer (CT) states from the carbazole-donor moiety to triazole-acceptor unity. However, the similar band with the low-energy shoulder at 363 nm and a tail up to 375 nm was recorded for triazole which was used as the reference (Figure 4a). Apparently, the LEBs of absorption spectra of **5a**–**5c** are due to the combination of *n*–π* transitions of the triazole-acceptor units and CT formed in ground states. If to consider the below-discussed exciplex formation for which CT in the ground state is not favorable,^52^ the LEBs of absorption spectra of **5a**–**5c** are mainly related to *n*–π* transitions of the triazole-acceptor units. It can be additionally noted that the UV/vis spectrum of the solution of compound **5c** exhibited a broader absorption band at 342 nm that apparently resulted from the overlap of several transitions toward various excited states. Absorption spectra of the solid samples were found to be similar to those of the dilute solutions, but the peaks were broader. The absorption maxima of the solid samples of compounds **5b** and **5c** were found to be slightly redshifted, in comparison to those of the corresponding dilute solutions (Figure 4a). No well-recognized effect of the donor position in the molecular structure on absorption spectra of compounds **5a**, **5b**, and **5c** was observed.

**Table 3:** Electroluminescent Parameters of White OLEDs

OLED structures: ITO/MoO_3_/TFB/emissive layer/TSPO1/TPBi/LiF:Al								
device	emissive layer	*V*_on_^a^(V)	max. brightness,cd/m^2^^b^	CE_max_, cd/A^c^	EQE_max_/EQE_1000_, %^d^	CIE (*x*; *y*)^e^	CRI^f^	*T*_C_, K^g^
**A11**	**5a**:SY-PPV:Ir(piq)_2_(acac) (98:1:1)	4.6	6813	9.6	3.4/2.6	(0.26, 0.28)	91	8711
**A12**	**5a**:SY-PPV:Ir(piq)_2_(acac) (97:1:2)	5	6059	7	3.2/2.6	(0.31, 0.34)	92	5349
**A15**	**5a**:SY-PPV:Ir(piq)_2_(acac) (94:1:5)	4.9	2245	3.6	1.3/0.8	(0.43, 0.36)	82	2358
**A51**	**5a**:SY-PPV:Ir(piq)_2_(acac) (94:5:1)	4.4	6162	8.7	3.8/2.7	(0.34, 0.45)	73	4828
**A52**	**5a**:SY-PPV:Ir(piq)_2_(acac) (93:5:2)	3.6	10882	18.4	7.1/6.2	(0.34, 0.41)	84	4632
**A55**	**5a**:SY-PPV:Ir(piq)_2_(acac) (90:5:5)	4.8	4389	3.6	1.8/1.6	(0.34, 0.38)	90	4435

a Turn-on voltage at luminance of 10 cd m^–2^.

b Maximum brightness.

c Maximum current efficiency.

d Maximum external quantum efficiency (EQE_max_) and EQE at 1000 cd/m^2^ (EQE_1000_).

e Commission Internationale de I’Eclairage (CIE) 1931 color coordinates.

f Color rendering index.

g Color temperature (CIE, CRI, and *T*_C_ values are related to EL spectra recorded at 10 V).

PL spectra of dilute toluene solutions of compounds **5a**, **5b**, and **5c** exhibited unstructured bands with the intensity maxima at ca. 480 nm (Figure 4b). The toluene solutions of compounds **5a**, **5b**, and **5c** demonstrated considerable increase of PL intensities after deoxygenation (Figure 4c). For compound **5b**, the ratio of PL intensities observed after and before evacuation was found to be of 2.2. Such increase of PL intensity under evacuation indicates participation of excited triplet states in emission of the compounds and possibly can be attributed to TADF.^53^ Indeed, PL decays of toluene solutions **5a**–**5c** were characterized by prompt and delayed components of the different intensities (Figure S1).

**Figure 5:**
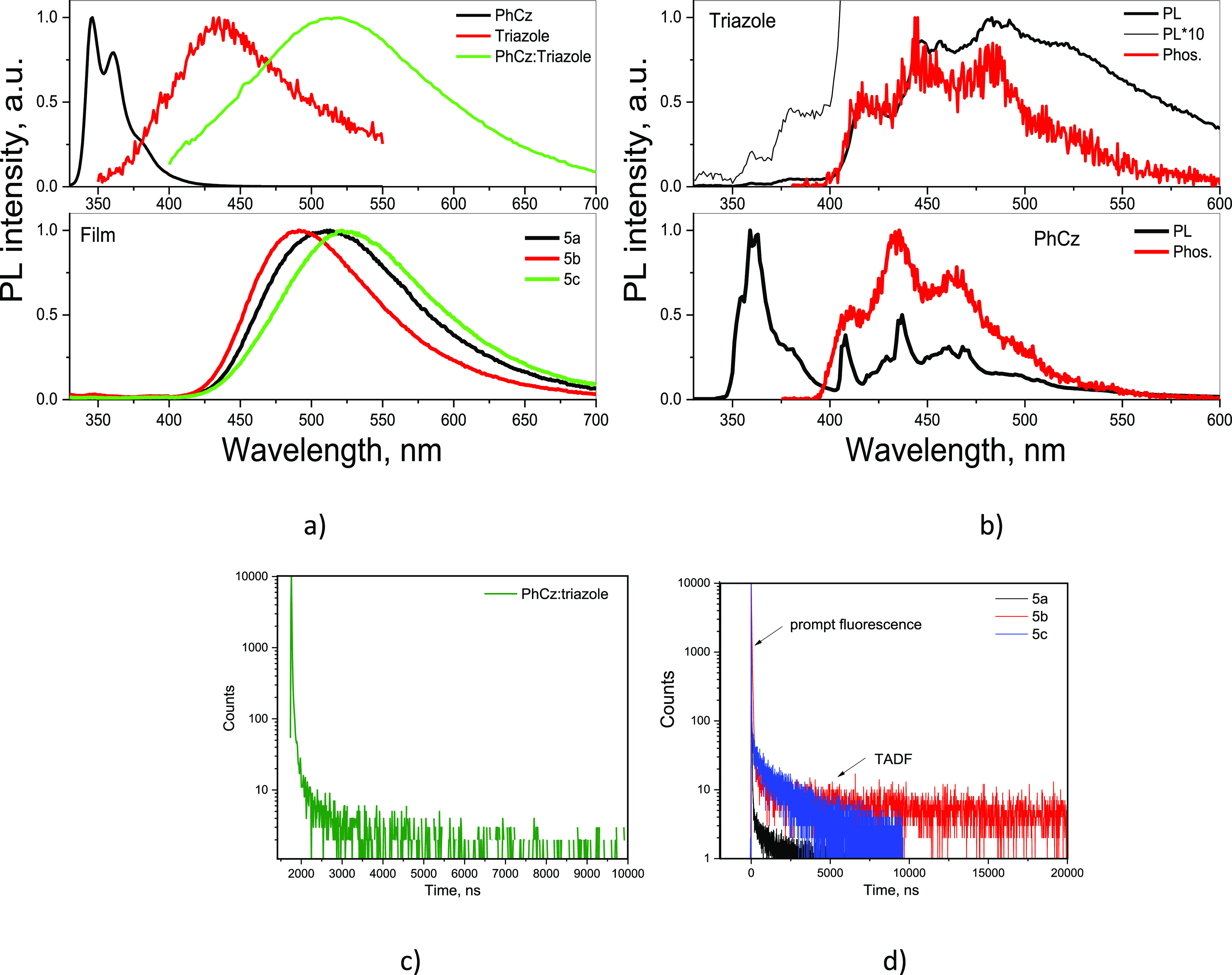
PL spectra of solid films and dilute THF solutions of exciplex in comparison to **5a**, **5b**, and **5c** (a); phosphorescence spectra of triazole and phenylcarbazole (b); PL decay curve of the exciplex film (c); PL decay curves of the **5a**, **5b**, and **5c** films (d). Excitation wavelengths of 350 nm.

PL spectra of dilute THF solutions of 5a-5c were slightly redshifted in comparison to the corresponding spectra of toluene solutions (Figure 4b). Such or even much stronger shifts are typical for donor–acceptor compounds due to the ICT nature of their emission.^40^ Compound **5c** containing two carbazole units was characterized by additional emission band in the UV region which can be assigned to recombination of local excited (LE) states of the carbazole moiety. LE nature of this additional band is evident because PhCz was characterized by emission by the similar PL spectrum (Figure 5a). It should be noted that PhCz was used as received without additional purification. We should note that, according to the previous study of carbazole derivatives exhibiting room temperature phosphorescence,^54−56^ the phosphorescence spectrum given the purchased PhCz shown in Figure 5 can be different from that of highly purified PhCz. Nevertheless, the onsets of phosphorescence spectra recorded at 77 K of the purchased PhCz and of highly purified PhCz should be practically the same as the similar onsets were previously observed for 9-(4-bromobenzyl)-9*H*-carbazole.^56^ Thus, the energy of local excited triplet states ^3^LE_D_ can be accurately determined from the onset of the recorded phosphorescene spectrum of the purchased PhCz. According to the onsets of phosphorescence spectra of PhCz and triazole (Figure 5b), ^3^LE_D_ is higher than ^3^LE_A_. This means that the energy level ^3^LE_D_ play a less important role in TADF than ^3^LE_A_.

To answer why compound **5c** is characterized by EL and CT emission bands in contrast to mainly CT emission observed for the similar compounds **5a** and **5b**, we assumed exciplex-like emission nature of **5a**–**5c** that is possible when formation of through-space CT (TSCT) states between electron-donating and electron-accepting moieties are predominant in the case of covalently bonded donor and acceptor units.^57−59^ Such TSCT states possess an emissive mechanism that cannot be distinguished from exciplexes; hence, such molecules are often referred to as “forming of intramolecular exciplexes”.^60^ Emission of the exciplex-forming systems is typically related to intermolecular exciplexes or to “intramolecular exciplexes” in some rare mentioned above cases.^60^

To prove the assumption of exciplex formation for **5a**–**5c**, the physical mixture of PhCz and triazole (50:50 wt %) was investigated (Figure 5a,b). In comparison to PL spectra of separate moieties, the mixture PhCz:triazole was characterized by a redshifted PL spectrum which was caused by exciplex formation. This observation supports the exciplex formation by **5a**–**5c** since exciplex formed by the physical mixture of the PhCz donor and triazole showed a very similar PL spectrum and PL decay to the PL spectra and PL decays of the solid samples of **5a**–**5c** (Figure 5). Thus, the emission of the solid layer of **5a**–**5c** is mainly of the exciplex nature in contrast to intramolecular TADF of through-bond conjugated D–A fragments. However, coexistence of both intermolecular exciplexes and “intramolecular exciplexes” is also possible in the solid layers **5a**–**5c**.

It should be noted that triazole demonstrated very weak fluorescence apparently because of the fast and efficient ISC (^1^LE_A_ → ^3^LE_A_) (Figure 5b). In addition, the energy of local excited triplet states, ^3^LE_A_ was practically the same as that of the intermolecular charge transfer state ^1^CT of exciplex systems (solid-state mixture of triazole and PhCz) which predetermines the efficient RISC pathway ^3^LE_A_ → ^1^CT of covalently-bonded exciplex-forming systems **5a**–**5c** as it was previously demonstrated for PCP-based exciplex systems.^40^

Due to the exciplex formation which is not very sensitive to polarity of the media, PL spectra of thin films of compounds **5a**–**5c** were found to be similar to those of the dilute toluene and THF solutions. However, PL decays of the films of **5a**–**5c** (Figure 5c) were characterized by considerably higher intensities of delayed fluorescence in comparison to those observed for toluene solutions of **5a**–**5c** (Figure S1) or for the solid films of 10% solid solutions of the investigated compounds in ZEONEX (Figure S2). This observation is the additional evidence of exciplex formation.^61^

The solid argument for the TADF nature of the delayed fluorescence of an emitter is a low value of singlet-triplet energy splitting (Δ*E*_ST_). To obtain Δ*E*_ST_ values for **5a**, **5b**, and **5c**, PL and phosphorescence spectra of the films of 10% solid solutions of the investigated compounds in ZEONEX were recorded at 77 K (Figure S3). The energies of the first singlet (S_1_) and triplet (T_1_) states are given in Table 2. Maxima of PL bands of compounds **5a**, **5b**, and **5c** were found to be not shifted in comparison to those observed at room temperature (Figure S3). Due to the similarities of the PL and phosphorescence spectra, the differences between energies of S_1_ and T_1_ were sufficiently small for all the studied compounds (ca. 0.01–0.02 eV) which are typical for exciplex-forming systems.^62^ This observation allows us to assume that the origin of emission of compounds **5a**, **5b**, and **5c** is TADF.^63^

**Figure 6:**
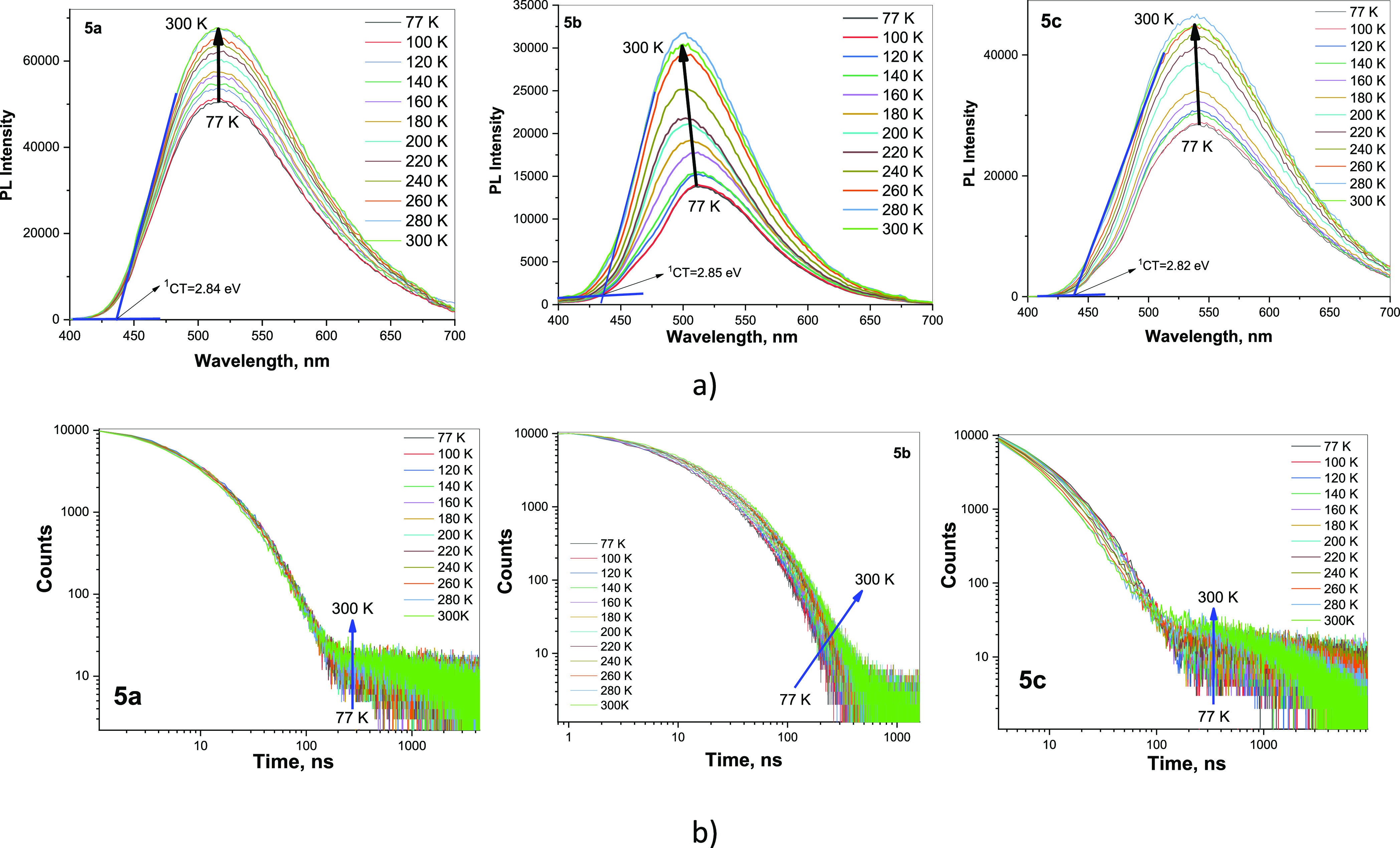
PL spectra (a) and PL decay curves (b) of the films of **5a**, **5b**, and **5c** recorded at different temperatures. The arrows are added for better guidance to the eyes.

To confirm TADF and to study the TADF mechanism in more detail, the films of **5a**, **5b**, and **5c** were additionally investigated by steady-state and time-resolved luminescence spectrometry at different conditions (Figures 6, S4–S6). The oxygen sensitivity of emission of **5a**, **5b**, and **5c** (Figure S4) shows that it involves triplet states via TADF as it was assumed above. The higher Δ*E*_ST_ values were obtained for the films of **5a**, **5b**, and **5c** than for their molecular dispersions in ZEONEX (Figure S5, Table 2). In addition, PL spectra and PL decay curves of the films of **5a**, **5b**, and **5c** were recorded at the different temperatures (Figure 6a,b). In contract to the previously reported observation for the PCP-based compounds which showed sub-microsecond TADF the thermal activation of which was undermined (emission intensity decreased) starting from ca. 200 K,^40^ the emission intensity of **5a**, **5b**, and **5c** gradually increased with increasing temperature from 77 to 280 K (marked in Figure 6a by thick arrows). This observation demonstrates the efficient thermal activation processes. It should be noted that the shapes of PL decay curves of **5a** and **5c** are similar to PL decay curves of conventional TADF emitters.^30,64^ They have the components of both prompt and delayed fluorescence. Meanwhile, the shapes of PL decay curves of **5b** are very similar to those of sub-microsecond TADF emitters. They practically have only delayed fluorescence components.^40^ We suppose that such sub-microsecond TADF of **5b** was observed due to the efficient population of ^3^LE_A_ via the ISC process and due to the similarity of ^3^LE_A_ and ^1^CT energy levels allowing efficient RISC. The mechanism of sub-microsecond TADF is not much discussed here since it was well described earlier.^40^ It is worth to note that compounds **5a**, **5b**, and **5c** can show combination of both conventional and sub-microsecond TADF. The most efficient sub-microsecond TADF was observed for compound **5b** because its ^1^CT level is closer to the ^3^LE_A_ level (Figures 5b and 6a). In other words, the most efficient conventional TADF was observed for compounds **5c** since its ^1^CT level is much lower than the ^3^LE_A_ level (Figures 5b and 6a). Apparently, because of the different combination of conventional and sub-microsecond TADF, different full widths at half-maxima (fwhm) were observed for the films of **5a**, **5b**, and **5c** (Figure S6). The narrowest PL spectrum with a fwhm of 99 nm was obtained for the films of compound **5b**.

PL quantum yields (PLQY) of the solutions and the films are given in Table 2. In the case of compounds **5a** and **5b** containing a single carbazole moiety, the PLQY values of the films were found to be slightly higher than those of the solutions. This observation is in very good agreement with the above-discussed exciplex-based emission mechanism of compounds **5a** and **5b**. In contrast, compound **5c** showed the lowest PLQY in the solid state because of the unbalanced number of donor and acceptor moieties (two-to-one in contrast to one-to-one in the case of **5a** and **5b**).

### 2.4 Solution-Processed Hybrid White Organic Light-Emitting Diodes

**Figure 7:**
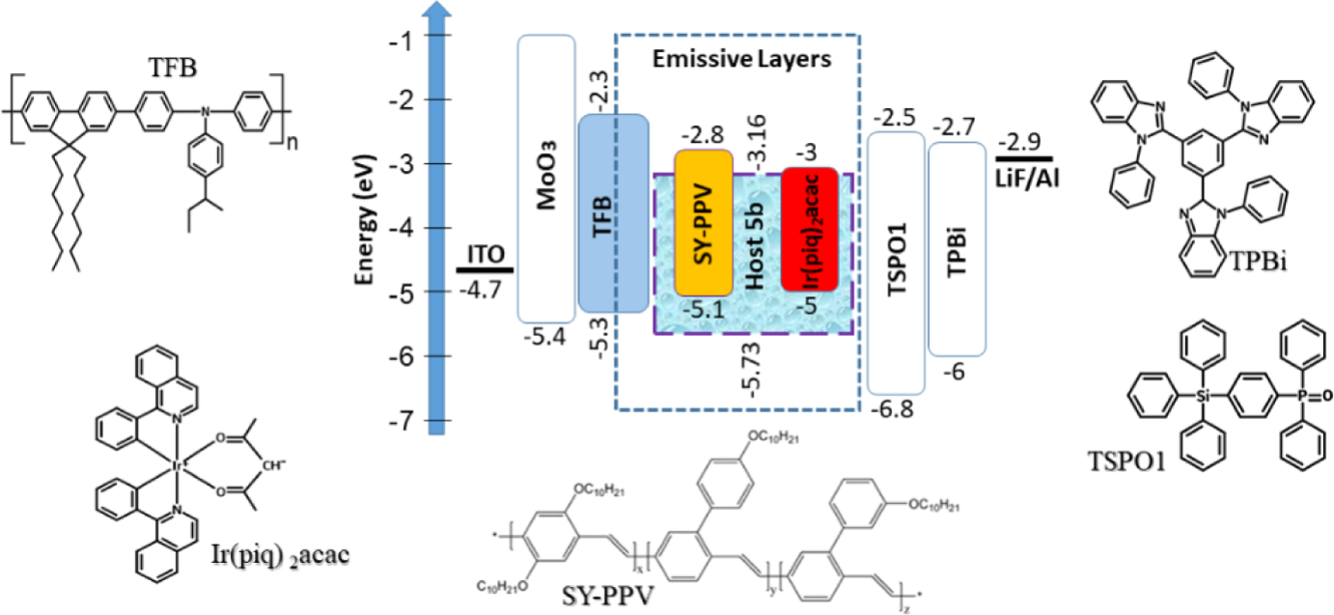
Visualized device structure with indication of energy levels of all functional layers and the molecular structures of the compounds used in the devices.

**Figure 8:**
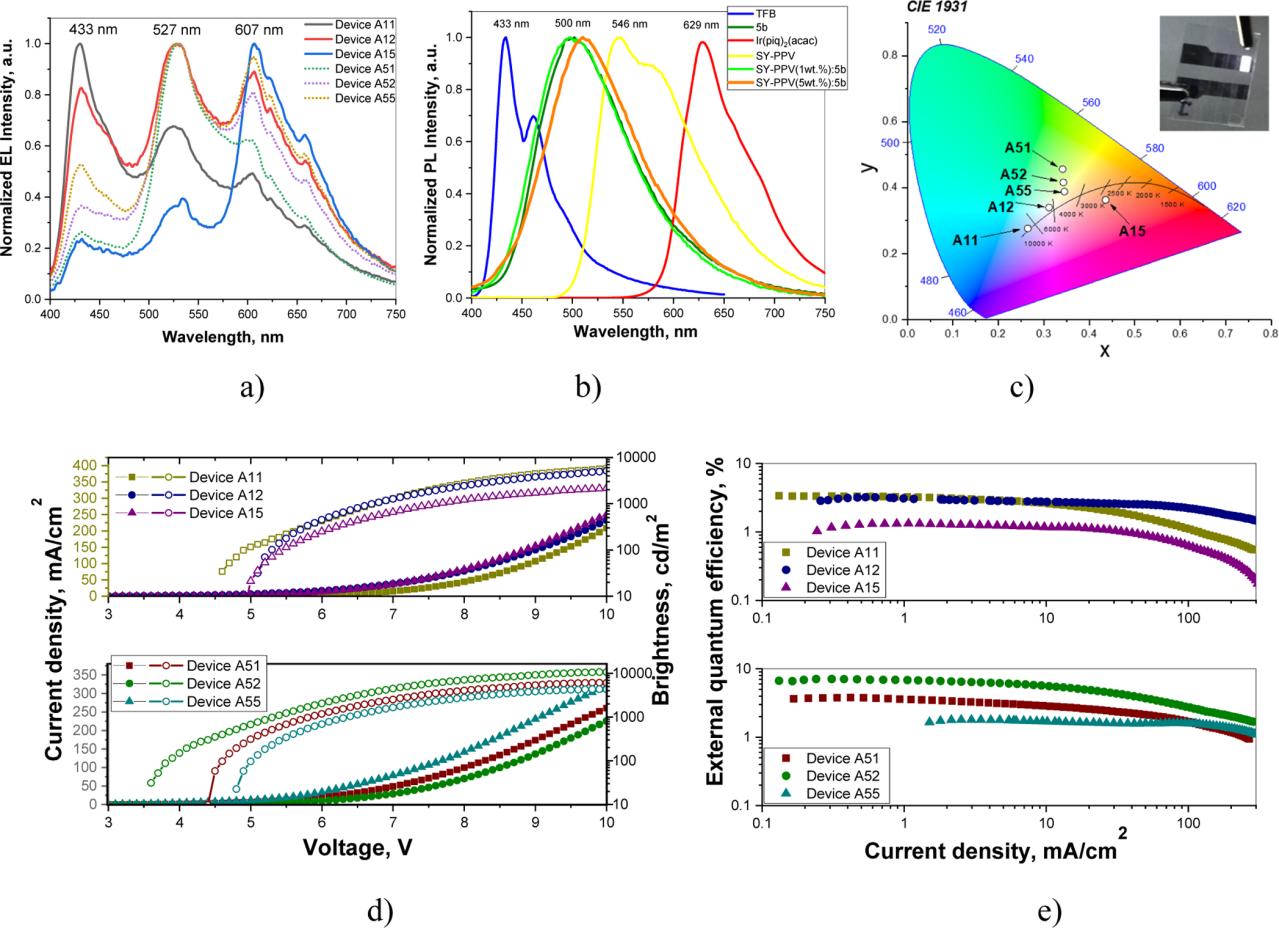
Normalized electroluminescence spectra recorded at 10 V (a); PL spectra of the host-emitter systems (b); CIE 1931 color diagram (c); current density/brightness versus applied voltage plots (d); and external quantum efficiency versus current density plots (e) for the studied OLEDs.

Taking into account the PL spectrum of compound **5b** which appeared in the greenish-blue region, this compound was used as a light-emitting host in hybrid WOLED structures. In addition, the selection of compound **5b** was determined by its better TADF performance than that of **5a** and **5c** as it is shown above (Figures 4–6). EL properties of the compound were investigated using the spin-coating method for the preparation of the emissive layer. Blue light-emitting poly (9,9-dioctylfluorene-*alt*-N-(4-*sec*-butylphenyl)-diphenylamine) (TFB), red light-emitting bis(1-phenylisoquinoline) (acetyl-lacetonate)iridium(III) (Ir(piq)_2_(acac)), and yellow light-emitting PPV copolymer Super Yellow (SY-PPV) were selected to obtain natural white emission with high color quality. The structures of OLEDs were as follows (Figure 7): ITO/MoO3 (1 nm)/TFB (30 nm)/**5b**:SY-PPV (*X* wt %):Ir(piq)_2_(acac) (*Y* wt %) (20 nm)/TSPO1 (8 nm)/TPBi (40 nm)/LiF(0.5 nm):Al(100 nm), where MoO_3_, TSPO1, and TPBi were employed for the preparation of the hole injection layer, hole/exciton blocking layer, and electron transporting layer, respectively. The layer of LiF was the electron injection layer and Al was the cathode. The effects of concentrations of Super Yellow and red phosphorescent emitter on the quality of white electroluminescence were investigated. Low concentrations of 1 and 5% and of 1, 2 and 5% were selected for Super Yellow and Ir(piq)_2_(acac), respectively, and EL spectra of devices A11, A12, A15, A51, A52, and A55 were recorded. Such concentrations of emitters in light-emitting layers with three components (one host and two emitters) were easily obtainable since the layers were fabricated by the spin-coating method. Figure 7 shows the chemical structures of the materials used for the fabrication of WOLEDs as well as the relative energy-level diagram. As it is shown in Figure 8a, EL spectra of the hybrid OLEDs were characterized by three emission bands observed at 430, 530, and 606 nm, which can be assigned to emissions of emitters TFB, SY-PPV, and Ir(piq)_2_(acac), respectively (Figure 8b). The band at 530 nm resulted from overlapping of emission of 5b and SY-PPV.

As it was expected, the highest intensity of the TFB emission band was observed in the electroluminescence spectrum of device A11 with the lowest concentrations of Super Yellow (1 wt %) and red emitter of Ir(piq)_2_(acac) (1 wt %) in the light-emitting layers. At the same time, the highest intensities of the Ir(piq)_2_(acac) emission band was observed in the electroluminescence spectrum of device A15 with the lowest concentrations of Super Yellow (1 wt %) and the highest concentration of the red phosphorescent emitter (5 wt %) (Figure 7). Due to the different concentrations of emitters, various characteristics of white electroluminescence [CIE1931 coordinates, CRI, and color temperature (*T*_C_)] were obtained for the hybrid OLEDs. The key performance parameters of the fabricated WOLEDs are shown in Figure 8 and summarized in Table 3. Figure 8c shows the CIE 1931 chromaticity diagram of the emission of the fabricated OLEDs. Almost all of the devices emitted light near the black body radiation locus. This reveals that they can be used as excellent lighting sources. The *T*_C_ values varied from a minimum of 2358 K to a maximum of 8711 K. The CRI values were between 73 and 92.

EL spectra of the devices showed some voltage-dependent character (Figure S7). The shifts of color coordinates were observed with increasing applied voltages due to the changes of the intensity of the respective EL peaks. This observation can be attributed to the energy transfer from the emitters exhibiting short-wavelength EL to the emitters exhibiting long-wavelength emission. The impressive CRI of 92 was observed for device A12 proving the best combination of intensities of blue, yellow, and red emissions in its EL spectrum with CIE1931 coordinates (0.31, 0.34) which were found to be the closest to those of the nature white (0.33, 0.33) and color temperature (*T*_C_) of 5349 K. The value of CRI is among the best values for hybrid white OLEDs observed up to now to the best of our knowledge.^22,65^

The resulting WOLEDs exhibited very relatively low turn-on voltages (Figure 8d). This observation proved that electrons and holes were efficiently injected and transported from electrodes and transport layers to the emissive layers and recombined in the light emitting layers. The turn on voltages of the devices ranged between 3.6 and 5 V at the luminance of 10 cd m^–2^ (Figure 8d, Table 3). Device A52 exhibited the highest maximum power efficiency of 10.7 lm/W, current efficiency of 18.4 cd/A, and quantum efficiency of 7.1% (Figures 8e and S8). This device also showed the lowest turn-on voltage of 3.6 with the highest brightness of 10,882 cd/m^2^. This generally means that the exciton recombination efficiency in device A52 was higher than in other fabricated devices, which may be ascribed to the considerable reduction of exciton annihilation and efficient triplet harvesting in phosphorescent emitter Ir(piq)_2_(acac), resulting in the improvement of efficiency of device A52.

## 3 Conclusions

Derivatives of carbazole and benzoyl-1*H*-1,2,3-triazole were synthesized employing Dimroth-type 1,2,3-triazole ring formation and Ullmann–Goldberg C–N coupling reactions. The compounds exhibited thermally activated delayed fluorescence caused by exciplex formations between electron-donating carbazole and newly developed electron-accepting moieties. It was confirmed by considerable increase of PL intensities of the solutions after deoxygenation and low singlet-triplet energy splitting of 0.01–0.2 eV. They were used as hosts in solution-processed white light-emitting diodes. On the basis of derivative of carbazole and benzoyl-1*H*-1,2,3-triazole, efficient solution-processed white light-emitting diodes were fabricated. The best device exhibited a maximum power efficiency of 10.7 lm/W, current efficiency of 18.4 cd/A, and external quantum efficiency of 7.1%. This device also showed a low turn-on voltage of 3.6 with a high brightness of 10,882 cd/m^2^.

## Supporting Information Available

Instruments, materials, methods, spectral data, and additional photophysical data (PDF)
