# Supplementary material for: Exciplex-Forming Systems of Physically Mixed and Covalently Bonded Benzoyl-1H-1,2,3-Triazole and Carbazole Moieties for Solution-Processed White OLEDs
Source: J Org Chem. 2022 Mar 4;87(6):4040–50. doi: 10.1021/acs.joc.1c02784 (PMC8938954; doi:10.1021/acs.joc.1c02784)
Supplement: Supplementary file 1 — jo1c02784_si_001.pdf [file jo1c02784_si_001.pdf]

Exciplex-forming systems of physically mixed and covalently bonded benzoyl-1H-1,2,3-triazole and carbazole moieties for solution processed white OLEDs

Mariia Stanitska[1,2], Malek Mahmoudi[1], Nazariy Pokhodylo[2], Roman Lytvyn[2], Dmytro Volyniuk[1], Ausra Tomkeviciene[1], Rasa Keruckiene[1], Mykola Obushak[2], Juozas Vidas Grazulevicius[1*]

[1]*Department of Polymer Chemistry and Technology, Kaunas University of Technology, Baršausko Str. 59, LT-51423, Kaunas, Lithuania*

[2]*Ivan Franko National University of Lviv, Kyryla i Mefodiya 6, Lviv, Ukraine*

Contents

Experimental Section S1
Instrumentation S5
Figures and tables S7
References S20

Experimental Section

Materials and methods

Materials used in this work include the molybdenum(VI) oxide (MoO_3), poly(9,9-dioctylfluorene-alt-N-(4-*sec*-butylphenyl)-diphenylamine) (TFB), bis(1-phenylisoquinoline)(acetylacetonate)iridium(III) ($Ir(piq)_2(acac)$), diphenyl-4-triphenylsilylphenyl-phosphine oxide (TSPO1), 2,2',2"-(1,3,5-benzinetriyl)-tris(1-phenyl-1-*H*-benzimidazole) (TPBi), Super Yellow light-emitting PPV copolymer and lithium fluoride (LiF) which were purchased from Sigma-Aldrich or Lumtec companies and used without additional purification. **Figure 7a** shows molecular structures of above organic materials.

1-Azido-4-fluorobenzene (**1**) was synthesized according to the reported procedure from the corresponding diazo salt [1,2].

1-(Bromoaryl)-4,4,4-trifluorobutane-1,3-dione[3] (**2a**) was synthesised according to the reported procedure [3,4].

1-(3-Bromophenyl)-4,4,4-trifluorobutane-1,3-dione (**2b**) and **1-(2,5-dibromophenyl)-4,4,4-trifluorobutane-1,3-dione** (**2c**) was synthesised according to the reported procedures [3].

* Corresponding author: Juozas Vidas Grazulevicius, *e-mail:* juozas.grazulevicius@ktu.lt

Synthesis of (Bromoaryl)(1-aryl-5-(trifluoromethyl)-1H-1,2,3-triazol-4-yl)methanones 3 (general procedure). A mixture of the corresponding 1-(bromoaryl)-4,4,4-trifluorobutane-1,3-dione **2** (3 mmol), 1-azido-4-fluorobenzene **1** (0.41 g, 3 mmol), and triethylamine (1.30 mL, 9 mmol) was heated at 70-75 °C for 5 h. After cooling to room temperature formed solid were triturate with isopropanol (10 ml) and filtered off. Dry on air to give target triazole **3**.

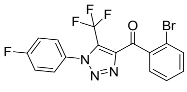

(2-Bromophenyl)(1-(4-fluorophenyl)-5-(trifluoromethyl)-1*H*-1,2,3-triazol-4-yl)methanone (3a)

Yield 0.91 g (73%); white solid; m.p.: 128–129 ºC;

^{1}H NMR (DMSO-d_6, 500 MHz): δ 7.90 – 7.78 (m, 3H), 7.71 (d, 1H J = 6.5 Hz), 7.65 – 7.49 (m, 4H).

^{13}C{^{1}H} NMR (DMSO-d6, 126 MHz): δ 186.8, 163.8 (d, $^1J_{C-F}$ = 249.5 Hz,), 145.0, 139.0, 133.9, 133.8, 131.8 (d, $^4J_{C-F}$ = 2.2 Hz), 131.4, 129.7 (q, $^2J_{C-F}$ = 42.3 Hz), 129.4 (d, $^3J_{C-F}$ = 9.4 Hz), 128.1, 119.9, 119.2 (q, $^1J_{C-F}$ = 270.8 Hz), 117.2 (d, $^2J_{C-F}$ = 23.6 Hz).

MS (m/z): 414, 416 (M$^+$+1);

Anal. calcd. for $C_{16}H_8BrF_4N_3O$: C, 46.40; H, 1.95; N, 10.15. Found: C, 46.47; H, 1.83; N, 10.21.

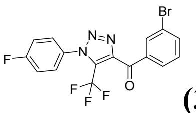

(3-Bromophenyl)(1-(4-fluorophenyl)-5-(trifluoromethyl)-1*H*-1,2,3-triazol-4-yl)methanone (3b)

Yield 0.98 g (79%); white solid; m.p.: 136–137 ºC;

^{1}H NMR (DMSO-d_6, 500 MHz): δ 8.27 (s, 1H), 8.11 (d, 1H, J = 6.5 Hz), 7.99 (d, 1H, J = 6.4 Hz), 7.86 (br.s, 2H), 7.62 (t, 1H, J = 6.8 Hz), 7.56 (t, 2H, J = 6.9 Hz).

^{13}C {^{1}H}NMR (DMSO-d$_6$, 126 MHz): δ 184.5, 163.8 (d, $^1J_{C-F}$ = 249.9 Hz,), 144.8, 138.0, 137.5, 133.3, 131.8 (d, $^4J_{C-F}$ = 1.9 Hz), 131.5, 130.2 (q, $^2J_{C-F}$ = 43.4 Hz), 129.7, 129.4 (d, $^3J_{C-F}$ = 9.3 Hz), 122.4, 120.3, 117.2 (d, $^2J_{C-F}$ = 23.5 Hz), 117.1 (q, $^1J_{C-F}$ = 270.0 Hz).

MS (m/z): 414, 416 (M$^+$+1);

Anal. calcd. for $C_{16}H_8BrF_4N_3O$: C, 46.40; H, 1.95; N, 10.15. Found: C, 46.47; H, 1.83; N, 10.21.

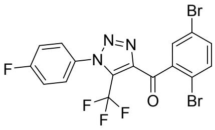

(2,5-Dibromophenyl)(1-(4-fluorophenyl)-5-(trifluoromethyl)-1*H*-1,2,3-triazol-4-yl)methanone (3c)

Yield 0.86 g (58%); white solid; m.p.: 172–173 ºC;

^{1}H NMR (DMSO-d_6, 500 MHz): δ 8.12 (s, 1H), 7.86 – 7.81 (m, 3H), 7.68 (d, 1H, J = 8.1 Hz), 7.54 (t, 2H, J = 7.9 Hz).

^{13}C {^{1}H}NMR (DMSO-d$_6$, 126 MHz): δ 186.0, 163.8 (d, $^1J_{C-F}$ = 249.7 Hz), 144.7, 138.1, 136.0,

132.8, 131.7 (d, $^4J_{C\text{-}F}$ = 2.0 Hz), 131.3, 129.9 (q, $^2J_{C\text{-}F}$ = 42.5 Hz), 129.4 (d, $^3J_{C\text{-}F}$ = 9.3 Hz), 126.3, 121.0, 119.1 (q, $^1J_{C\text{-}F}$ = 271.3 Hz), 117.2 (d, $^2J_{C\text{-}F}$ = 23.6 Hz).

MS (m/z): 492, 494, 496 (M^{+}+1);

Anal. calcd. for $C_{16}H_7Br_2F_4N_3O$: C, 38.98; H, 1.43; N, 8.52. Found: C, 38.81; H, 1.37; N, 8.71.

Synthesis of ((9*H*-carbazol-9-yl-)phenyl)(1-(4-fluorophenyl)-5-(trifluoromethyl)-1*H*-1,2,3-triazol-4-yl)methanones 5 (general procedure).

In a Schlenk flask (25 mL) bromocontaining triazoles **3a,b** (278 mg, 0.671 mmol), carbazole **4** (0.2 g, 1.197 mmol), potassium carbonate (0.2 g, 1.45 mmol), copper powder (10 mg, 0.156 mmol), copper(I)chloride (10 mg, 0.100 mmol), 1,10-phenantroline (20 mg, 0.12 mmol) and xylene (7.0 mL) were placed. In case of triazole **3c** (331 mg, 0.671 mmol) loadings were increased twice: carbazole **4** (0.4 g, 2.394 mmol), potassium carbonate (0.4 g, 2.9 mmol), copper powder (20 mg, 0.312 mmol), copper(I)chloride (20 mg, 0.200 mmol), 1,10-phenantroline (40 mg, 0.24 mmol) and xylene (10 ml). The reaction was performed under gentle stirring and refluxing (oil bath temperature ≈ 150°C) for 36-48 hours (TLC control). After reaction completion, mixture was filtrated through a small pad of celite. All volatiles were removed in vacuum. The residue was purified by column chromatography on silica gel. Obtained compounds **5** additionally were recrystallized from appropriate solvents.

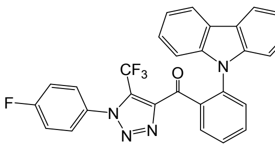

(2-(9*H*-Carbazol-9-yl)phenyl)(1-(4-fluorophenyl)-5-(trifluoromethyl)-1*H*-1,2,3-triazol-4-yl)methanone (5a)

Yield 161 mg (48%); light-yellow crystals; m.p.: 169-170 ºC; eluent for column chromatography – DCM:Hex (1:1); recrystallized from hexane.

^{1}H NMR ($CDCl_3$, 400 MHz): δ 8.09 (d, 1H, J = 7.6 Hz), 7.98 (d, 2H, J = 7.7 Hz), 7.89 (t, 1H, J = 7.2), 7.76 (t, 1H, J = 7.6 Hz), 7.66 (d, 1H, J = 7.8 Hz), 7.42 (t, 2H, J = 7.6 Hz), 7.35 – 7.25 (m, 4H), 7.13 (t, 2H, J = 8.4 Hz), 6.85 (dd, 2H, J = 8.6, 4.6 Hz).

^{13}C{^{1}H} NMR ($CDCl_3$, 101 MHz): δ 186.6, 163.7 (d, $^1J_{C\text{-}F}$ = 252.5 Hz), 144.8, 140.8, 137.4, 137.2, 134.1, 131.1, 130.9 (q, $^2J_{C\text{-}F}$ = 43.7 Hz), 130.6 (d, $^4J_{C\text{-}F}$ = 4.0 Hz), 129.0, 128.7, 127.7 (d, $^3J_{C\text{-}F}$ = 9.3 Hz), 126.3, 122.6, 120.5, 119.5, 116.3 (d, $^2J_{C\text{-}F}$ = 23.4 Hz), 115.1 (q, $^1J_{C\text{-}F}$ = 272.3 Hz), 110.5.

^{19}F NMR ($CDCl_3$, 376 MHz): δ -57.24, -108.38.

HRMS (ESI-TOF) m/z: $[M+H]^+$ Calcd for $C_{28}H_{17}F_4N_4O$ 501.1333; Found 501.1338.

IR ν_{max} (KBr) cm^{-1}: 3081 (–C–H Ar), 1682, 1600, (C=O); 1335, 1316, 1276 (–C–N–); 1195, 1170 (–CF_3); 1104, 1091 (–C–F).

Anal. calcd. for $C_{28}H_{16}F_4N_4O$: C, 67.20; H, 3.22; N, 11.20. Found: C, 67.15; H, 3.20; N, 11.25.

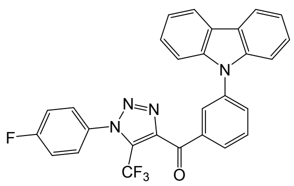

(3-(9*H*-Carbazol-9-yl)phenyl)(1-(4-fluorophenyl)-5-(trifluoromethyl)-1*H*-1,2,3-triazol-4-yl)methanone (5b)

Yield 198 mg (59%); white needles; m.p.: 176-177 ºC; eluent for column chromatography – DCM:Hex (1:1); recrystallized from ethanol.

^{1}H NMR ($CDCl_3$, 400 MHz): δ 8.43 (t, 1H, J = 1.8 Hz), 8.32 (dt, 1H, J = 7.8, 1.1 Hz), 8.19 (d, 2H, J = 7.8 Hz), 7.94 (ddd, 1H, J = 7.9, 1.1, 0.8 Hz), 7.84 (t, 1H, J = 7.8 Hz), 7.59 – 7.52 (m, 4H), 7.48 (dt, 2H, J = 7.6, 1.0 Hz), 7.37 – 7.29 (m, 4H).

^{13}C {^{1}H}NMR ($CDCl_3$, 101 MHz): δ 184.2, 161.4 (d, $^1J_{C\text{-}F}$ = 251.5 Hz), 145.6, 140.6, 138.3, 137.6, 132.5, 131.3 (d, $^4J_{C\text{-}F}$ = 4.0 Hz), 130.3 (q, $^2J_{C\text{-}F}$ = 43.6 Hz), 129.3, 127.8 (d, $^3J_{C\text{-}F}$ = 9.3 Hz), 126.2, 123.6, 120.4, 119.0 (q, $^1J_{C\text{-}F}$ = 270.8 Hz), 116.9 (d, $^2J_{C\text{-}F}$ = 23.5 Hz), 109.7.

^{19}F NMR ($CDCl_3$, 376 MHz): δ -56.11, -107.71.

HRMS (ESI-TOF) m/z: [M+H]$^+$ Calcd for $C_{28}H_{17}F_4N_4O$ 501.1333; Found 501.1342.

IR ν_{max} (KBr) cm^{-1}: 3124, 3078, 3059 (–C–H Ar), 1678, 1595, (C=O); 1337, 1316, 1290 (–C–N–); 1187, 1157 (–CF_3); 1096, 1047 (–C–F).

Anal. calcd. for $C_{28}H_{16}F_4N_4O$: C, 67.20; H, 3.22; N, 11.20. Found: C, 67.24; H, 3.30; N, 11.14.

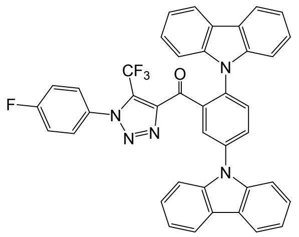

(2,5-Di(9*H*-carbazol-9-yl)phenyl)(1-(4-fluorophenyl)-5-(trifluoromethyl)-1*H*-1,2,3-triazol-4-yl)methanone (5c)

Yield 214 mg (48%); yellow microcrystals; m.p.: 253–254 ºC; eluent for column chromatography – DCM-Hex (2:1); recrystallized from ethanol.

^{1}H NMR (DMSO-d_6, 400 MHz): δ 8.36 (d, 1H, J = 8.2 Hz, 1H), 8.27 (d, 2H, J = 7.6 Hz), 8.19 – 8.11 (m, 3H), 8.04 (s, 1H), 7.73 (d, 2H, J = 8.2 Hz), 7.53 (t, 2H, J = 7.6 Hz), 7.48 – 7.34 (m, 8H), 7.27 (t, 2H, J = 7.2 Hz,), 7.21 (dd, 2H, J = 8.0, 4.6 Hz).

^{13}C {^{1}H}NMR (DMSO-d_6, 101 MHz): δ 185.5, 161.8 (d, $^1J_{C\text{-}F}$ = 250.9 Hz), 144.7, 143.1, 140.9, 140.2, 138.5, 135.0, 133.6, 131.1, 129.4 (q, $^2J_{C\text{-}F}$ = 43.1 Hz), 128.8 (d, $^3J_{C\text{-}F}$ = 9.3 Hz), 127.1, 127.1, 127.0, 126.8, 124.0, 123.0, 121.4, 121.1, 120.9, 120.5, 118.5 (q, $^1J_{C\text{-}F}$ = 273.8 Hz), 117.1 (d, $^2J_{C\text{-}F}$ = 23.9 Hz), 110.6, 110.4.

^{19}F NMR (DMSO-d_6, 376 MHz): δ -56.33, -108.67.

HRMS (ESI-TOF) m/z: [M]$^+$ Calcd for $C_{40}H_{24}F_4N_5O$ 666.1911; Found 666.1922.

IR ν_{max} (KBr) cm^{-1}: 3079, 3055, 3022 (–C–H Ar), 1679, 1600, (C=O); 1334, 1312, 1266 (–C–N–); 1148, 1123 ($–CF_3$); 1085, 1048(–C–F).

Anal. calcd. for $C_{40}H_{23}F_4N_5O$: C, 72.18; H, 3.48; N, 10.52. Found: C, 72.11; H, 3.43; N, 10.70.

Instrumentation

^1H and ^{13}C NMR spectra were recorded on Varian Unity Plus 400 (400 and 101 MHz, respectively) and Bruker Avance 500 (500 and 126 MHz, respectively) spectrometers in DMSO-d_6 solutions, using TMS or the residual peaks of the solvent (2.50 ppm for ^1H nuclei and 39.5 ppm for ^{13}C nuclei) as internal references. HRMS analysis was performed using Bruker Daltonics - maXis 4G - UHR-TOF, calibrated with Na format. Mass spectral analysis was performed using an Agilent 1100 series LC/MSD with API-ES/APCI mode (200 eV). Elemental analysis was performed using a Carlo Erba 1106 instrument. Melting points were determined on a Mel-Temp melting point apparatus. IR spectra were recorded on Bruker VERTEX 70 FT-IR spectrometer.

UV-visible absorption spectra of dilute (10^{-5} M) solutions and neat films were recorded on the UV–VIS–NIR Bruker spectrophotometer. The ground-state geometries were optimized by using the B3LYP (Becke three parameters hybrid functional with Lee-Yang-Perdew[5] correlation) functional at 6-31G (d, p) level in vacuum with the Gaussian[6] program. Firstly, the equilibrium conformer search at the ground state was performed by using the MMFF (Molecular mechanics force fields) method, and then this geometry was used for further optimization. Cyclic voltammetry measurements were performed using a platinum working electrode (a disk with the diameter of 2 mm) in a three-electrode cell of Autolab Type potentiostat-galvanostat. The measurements were carried out for the solutions in dry dichloromethane containing 0.1 M tetrabutylammonium hexafluorophosphate at 25 °C. The scan rate was of 50 mV/s while the sample concentration was of 10^{-3} M. The potentials were measured against silver as a quasi-reference electrode. Platinum wire was used as a counter electrode. The potentials were calibrated with the standard ferrocene/ferrocenium (Fc/Fc^+) redox system.[7] Photoluminescence (PL) spectra, photoluminescence quantum yield (PLQY) and excited state lifetime of dilute solutions (10^{-5} M) or solid films of the compounds were investigated with Edinburgh Instruments FLS980 spectrometer at room temperature and 77 ˚K. Thin solid films for measurement of UV/VIS and PL spectra were prepared by drop casting 2 mg/ml solutions of the compounds in toluene on the pre-cleaned quartz substrates. Liquid nitrogen cryostat Optistat DN2 was used for the characterization of photophysical properties of the samples and measuring temperature-dependent experiments (77 - 300 ˚K) under inert nitrogen atmosphere (N_2), while measurements at room temperature were recorded in vacuum in the same cryostat. FLS980 integrating sphere was used for recording the values of photoluminescence quantum yields by an absolute method at room temperature. FLS980 and a PicoQuant LDH-D-C-375 laser with the excitation wavelength of 374 nm of a beam were used to record PL decay curves.

Phosphorescence spectra of THF solutions were recorded at 77 ˚K with the delay time after excitation (300 nm) exceeding 50 ms. Luminance-voltage and Current density-voltage characteristics were recorded using a sourcemeter Keithley 2400C and PH100-Si-HA-D0 photodiode connected to the PC-Based power and energy monitor 11S-LINK in the air without passivation quickly after the fabrication of the device. Brightness measurements were performed using a calibrated photodiode. Electroluminescence (EL) spectra were recorded with an UV–VIS–NIR Avantes (AvaSpec-2048XL) spectrophotometer. CIE coordinates, color rendering index (CRI) and colour temperatures of samples were calculated by using EL spectra and device efficiencies were estimated from the luminance, EL spectrum and current density.

OLED fabrication

Electroluminescent devices with the structures of ITO/MoO_3 (1 nm)/ TFB (30 nm)/ **5a**:SY:Ir(piq)$_2$(acac) (20 nm) / TSPO1 (8 nm)/TPBi (40 nm)/LiF(0.5 nm):Al(100 nm) were fabricated by a spin-coating/evaporation hybrid method. The devices ware deposited on the glass substrates from Ossila with pre-patterned bottom indium tin oxide (ITO) electrode. The top Al electrode and vacuum-deposited organic layers were deposited using appropriate shadow masks (also from Ossila). In such case, four OLED pixels with size of 4.5 mm^2 were obtained per one substrate. TFB and the second light-emitting (**5a**:SY:Ir(piq)$_2$(acac)) layers were spin-coated while the other layers were vacuum-evaporated. Whole vacuum-evaporated layers fabricated via the physical vacuum deposition (PVD) technology were fabricated. During the device fabrications, the vacuum chamber was always kept at the pressure of 2×10^{-6} mBar. All of the vacuum-evaporated layers were deposited at a rate of 1 Å s^{-1} except the LiF layer which was fabricated at a rate of 0.1 Å s^{-1}. Emissive layers were deposited from toluene solutions (4 mg/ml) of the corresponding compound mixtures *inside* a *glove box* under inert gas atmosphere. After spin-coating of TFB and other light emitting layer, the samples were thermally annealed at 90 °C for 30 min (in case of TFB layer) or 180 °C for 40 min (in case of second emissive layer) using electric hot plate.

Figures and tables

^{1}H NMR (DMSO-d_6, 500 MHz) ((2-bromophenyl)(1-(4-fluorophenyl)-5-(trifluoromethyl)-1*H*-1,2,3-triazol-4-yl)methanone (3a):

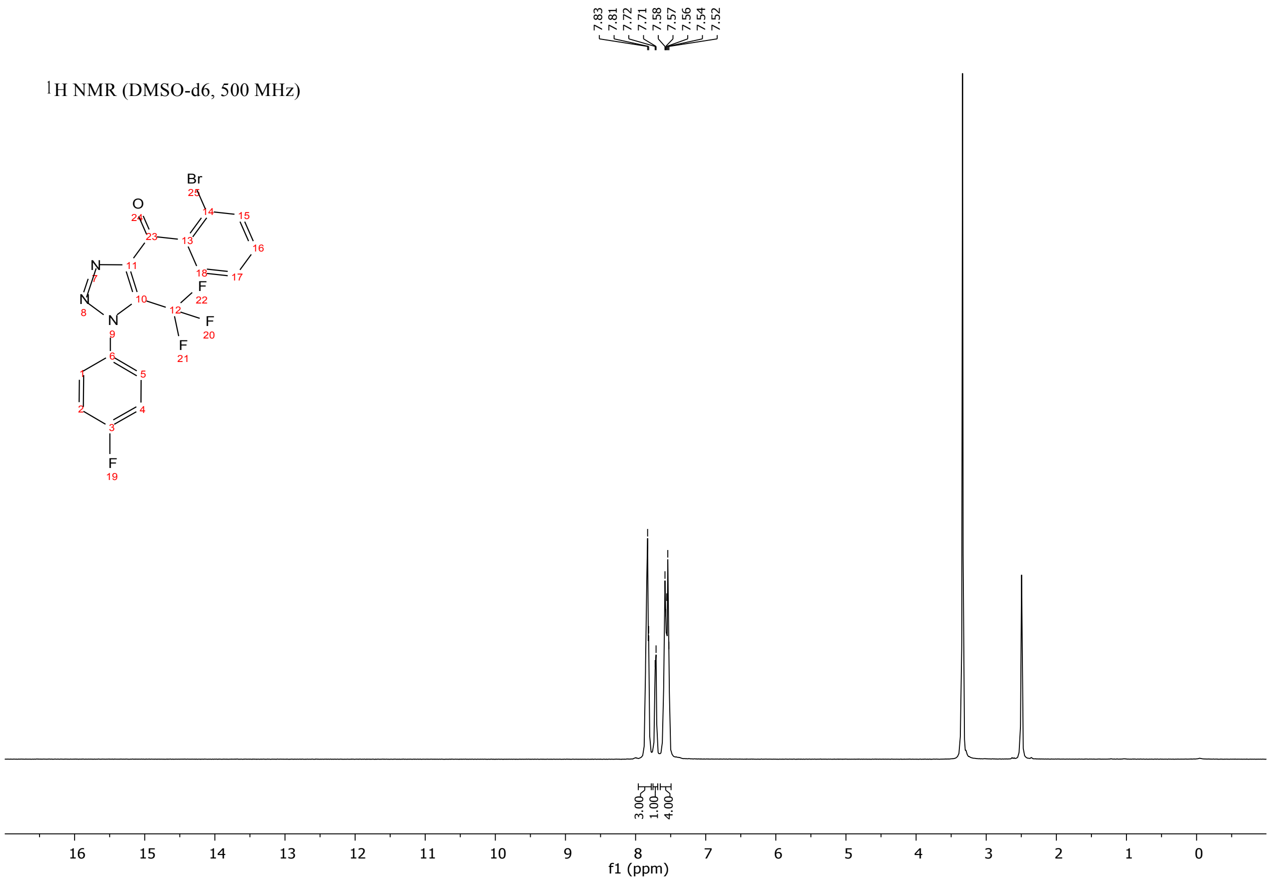

^{13}C{^{1}H} NMR (DMSO-d_6, 126 MHz) ((2-bromophenyl)(1-(4-fluorophenyl)-5-(trifluoromethyl)-1*H*-1,2,3-triazol-4-yl)methanone (3a):

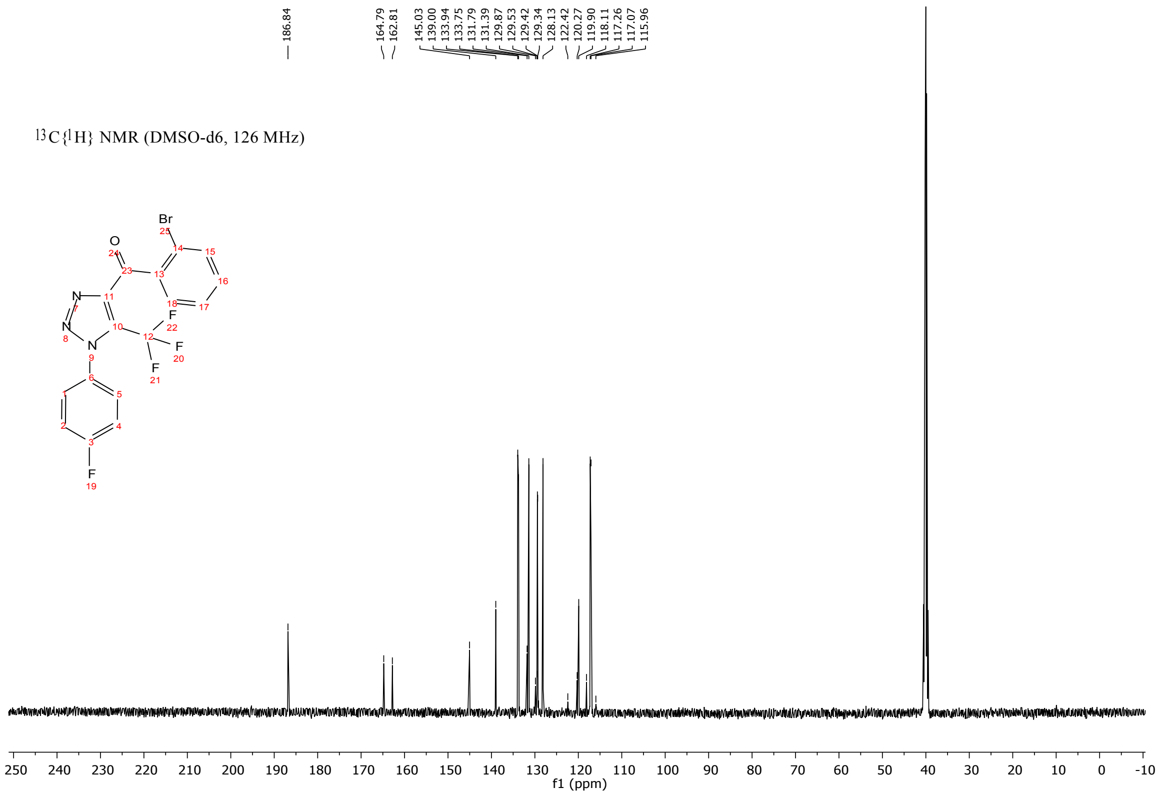

^{1}H NMR (DMSO-*d*$_6$, 500 MHz) ((3-bromophenyl)(1-(4-fluorophenyl)-5-(trifluoromethyl)-1*H*-1,2,3-triazol-4-yl)methanone (3b):

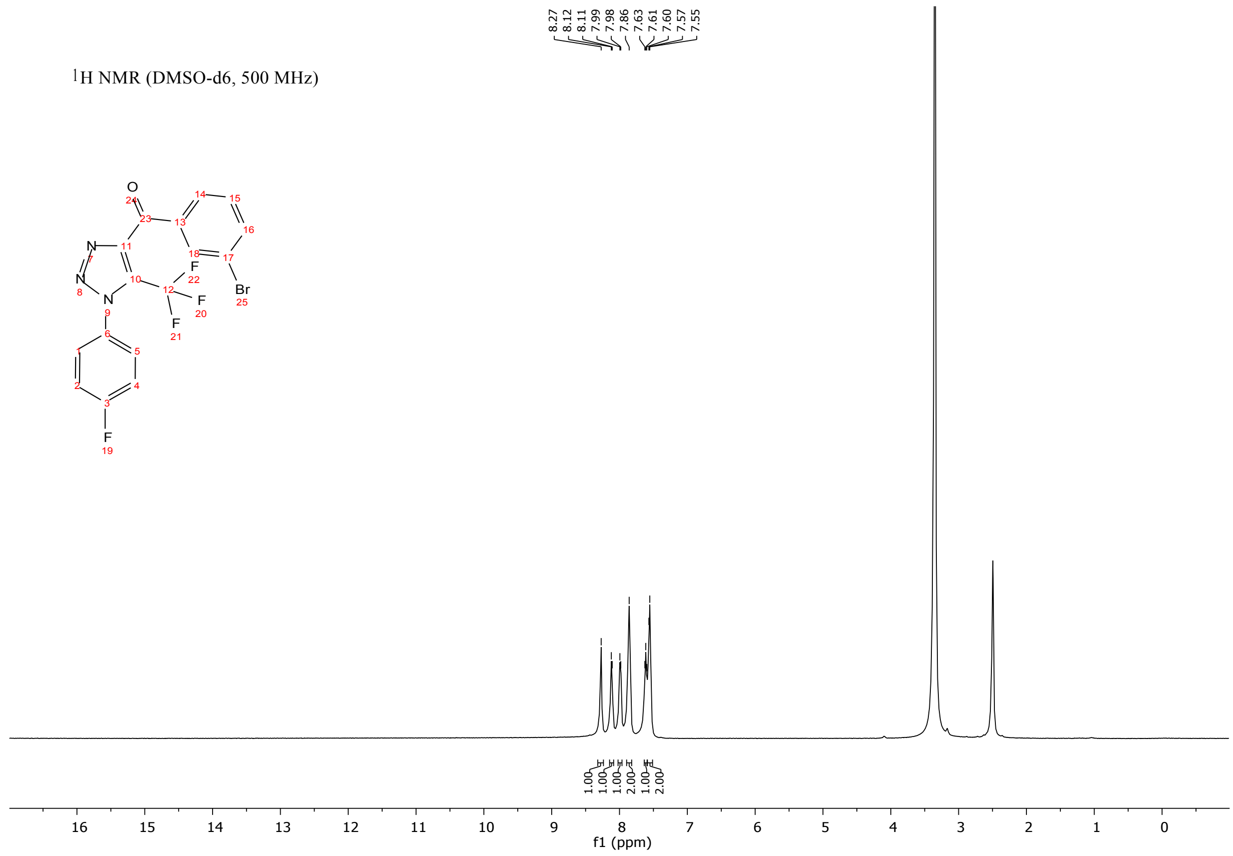

$^{13}C\{^{1}H\}$ NMR (DMSO-d_6, 126 MHz) ((3-bromophenyl)(1-(4-fluorophenyl)-5-(trifluoromethyl)-1*H*-1,2,3-triazol-4-yl)methanone (3b):

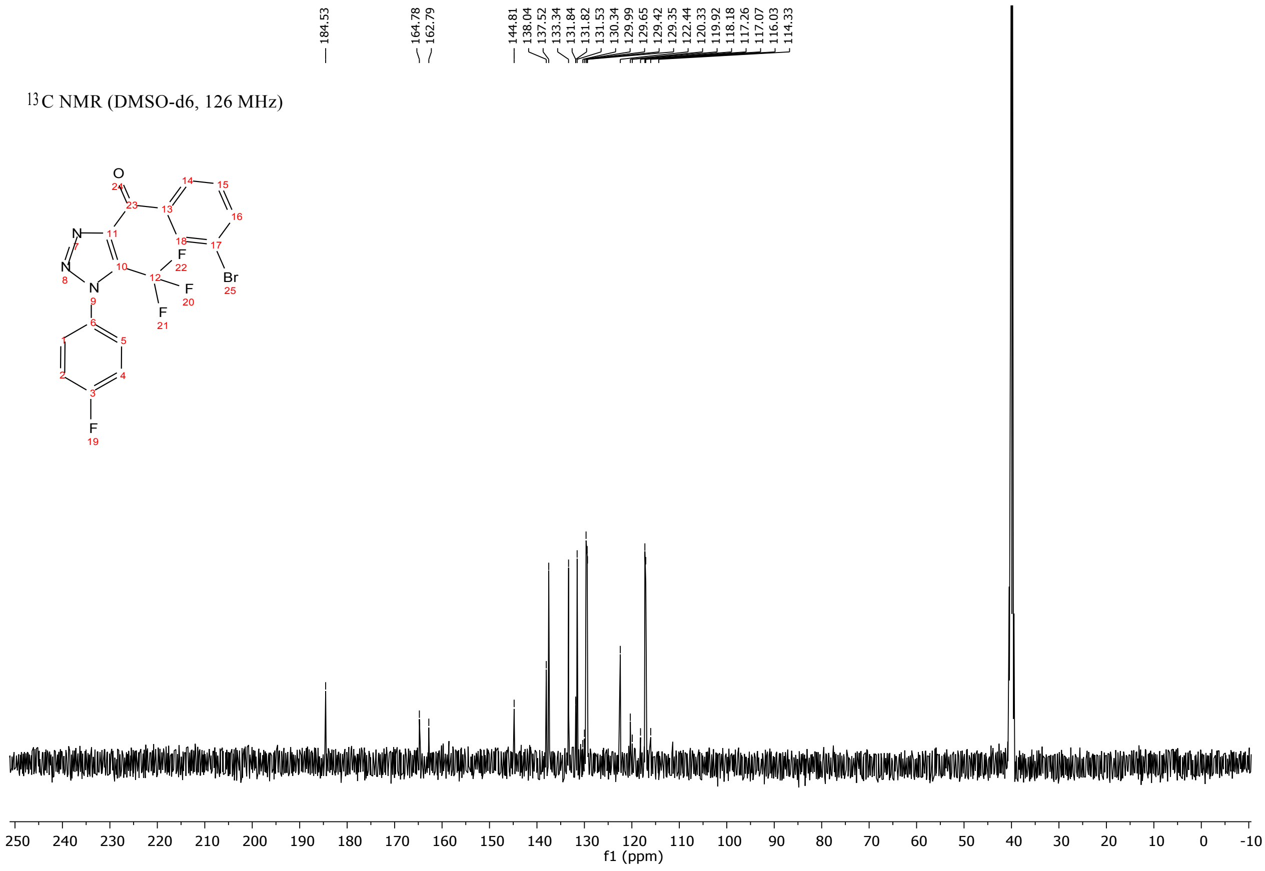

^{1}H NMR (DMSO-d_6, 500 MHz) (2,5-dibromophenyl)(1-(4-fluorophenyl)-5-(trifluoromethyl)-1*H*-1,2,3-triazol-4-yl)methanone (3c):

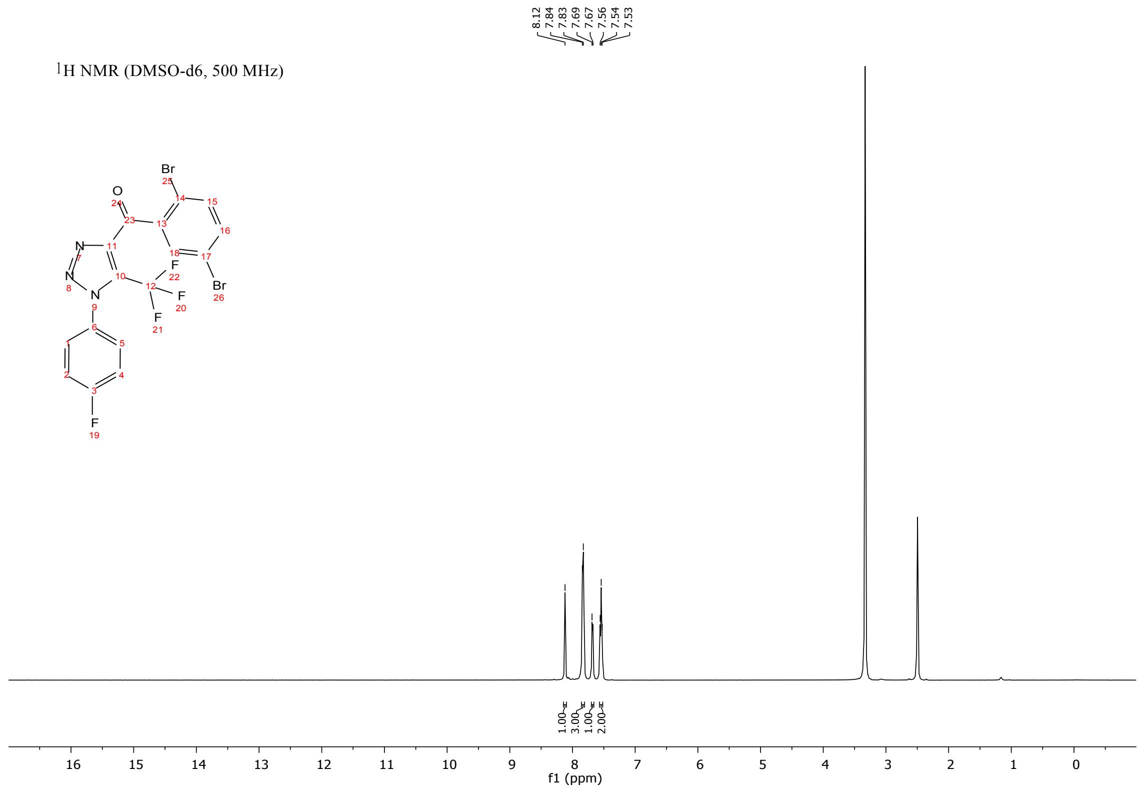

$^{13}C\{^{1}H\}$ NMR (DMSO-d_6, 126 MHz) (2,5-dibromophenyl)(1-(4-fluorophenyl)-5-(trifluoromethyl)-1*H*-1,2,3-triazol-4-yl)methanone (3c):

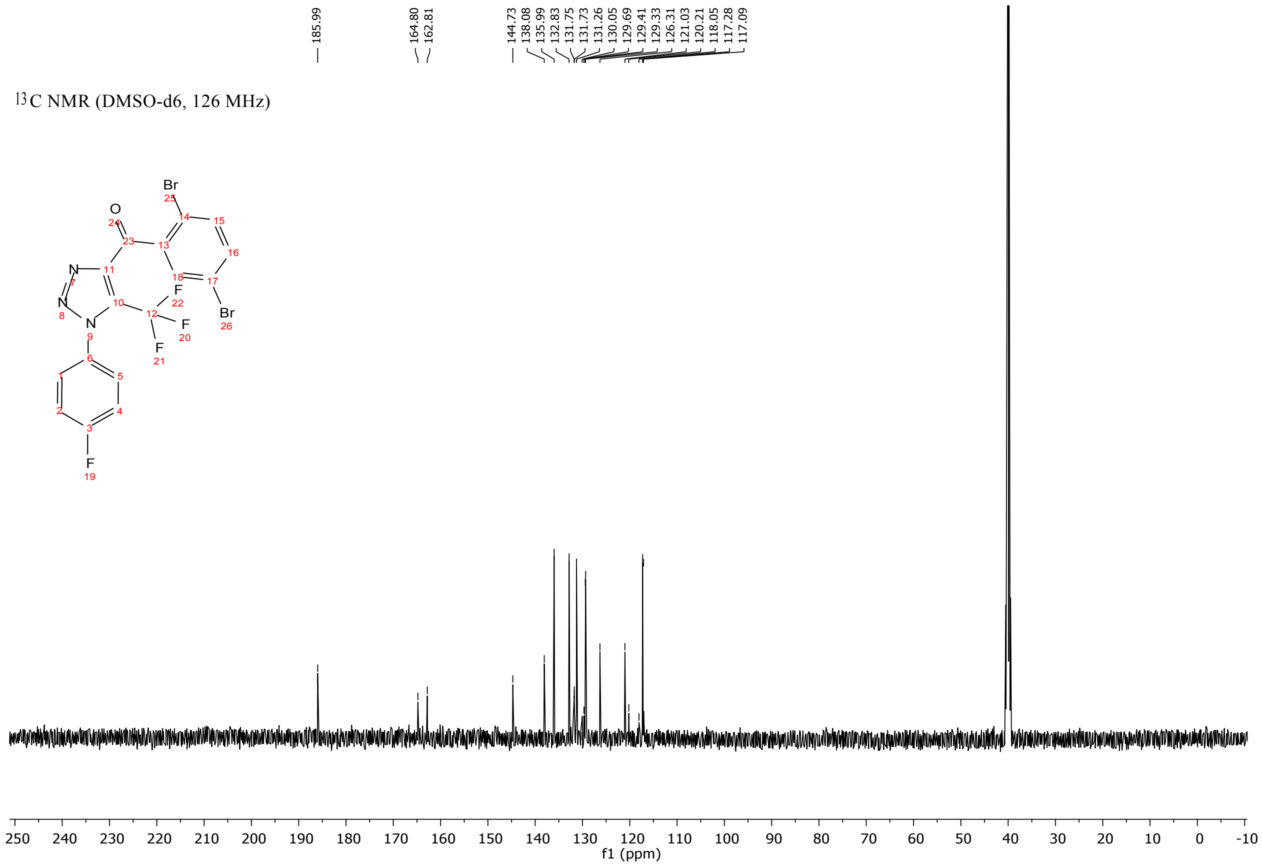

^{1}H NMR ($CDCl_3$, 400 MHz) (2-(9*H*-carbazol-9-yl)phenyl)(1-(4-fluorophenyl)-5-(trifluoromethyl)-1*H*-1,2,3-triazol-4-yl)methanone (5a):

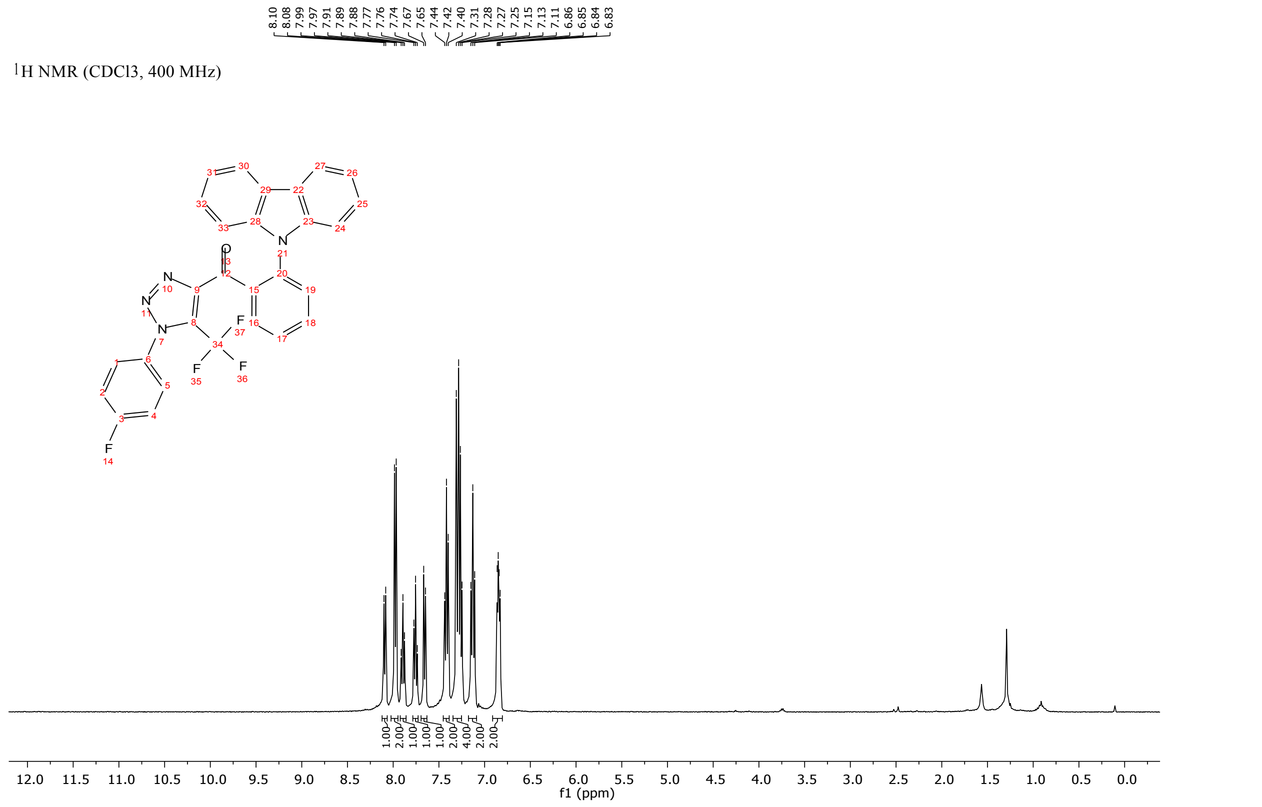

$^{13}C\{^{1}H\}$ NMR ($CDCl_3$, 101 MHz) (2-(9*H*-carbazol-9-yl)phenyl)(1-(4-fluorophenyl)-5-(trifluoromethyl)-1*H*-1,2,3-triazol-4-yl)methanone (5a):

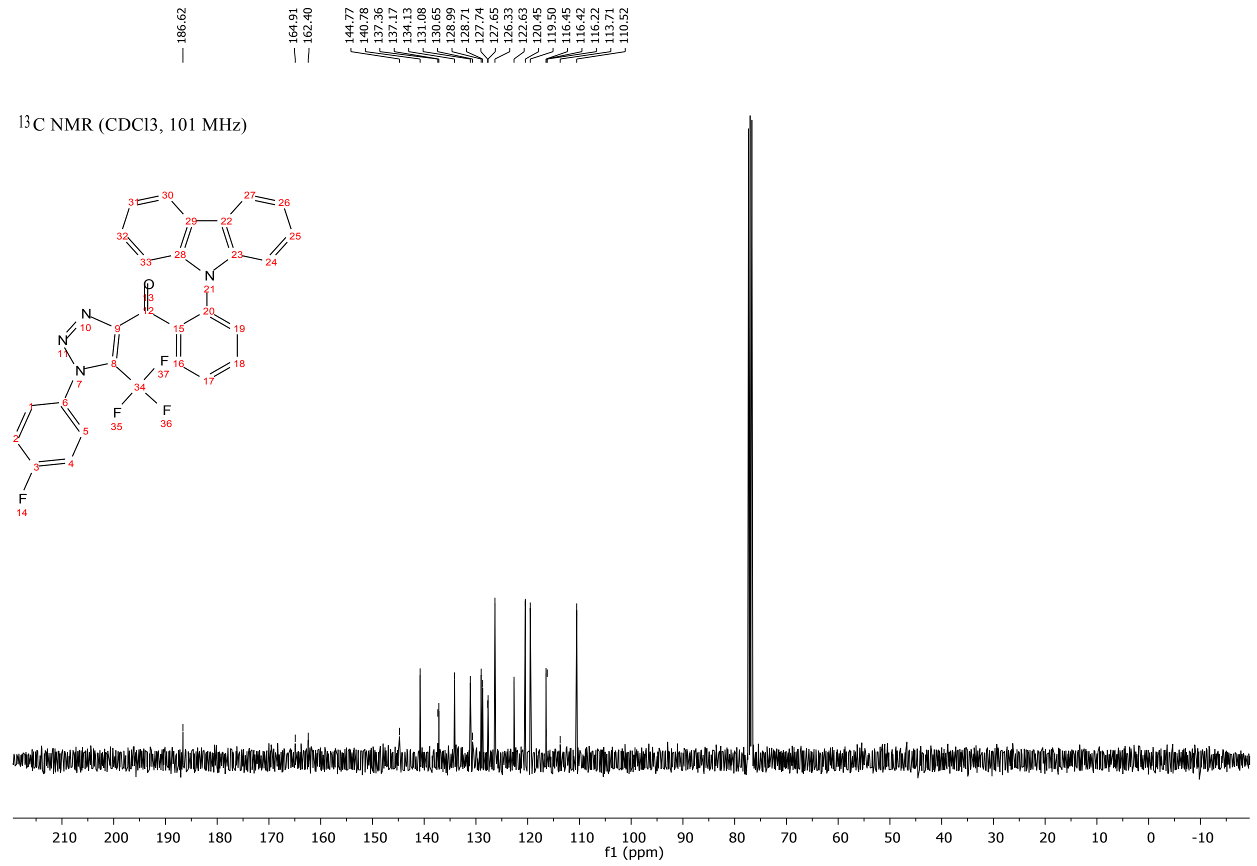

^{19}F NMR ($CDCl_3$, 376 MHz) (2-(9*H*-carbazol-9-yl)phenyl)(1-(4-fluorophenyl)-5-(trifluoromethyl)-1*H*-1,2,3-triazol-4-yl)methanone (5a):

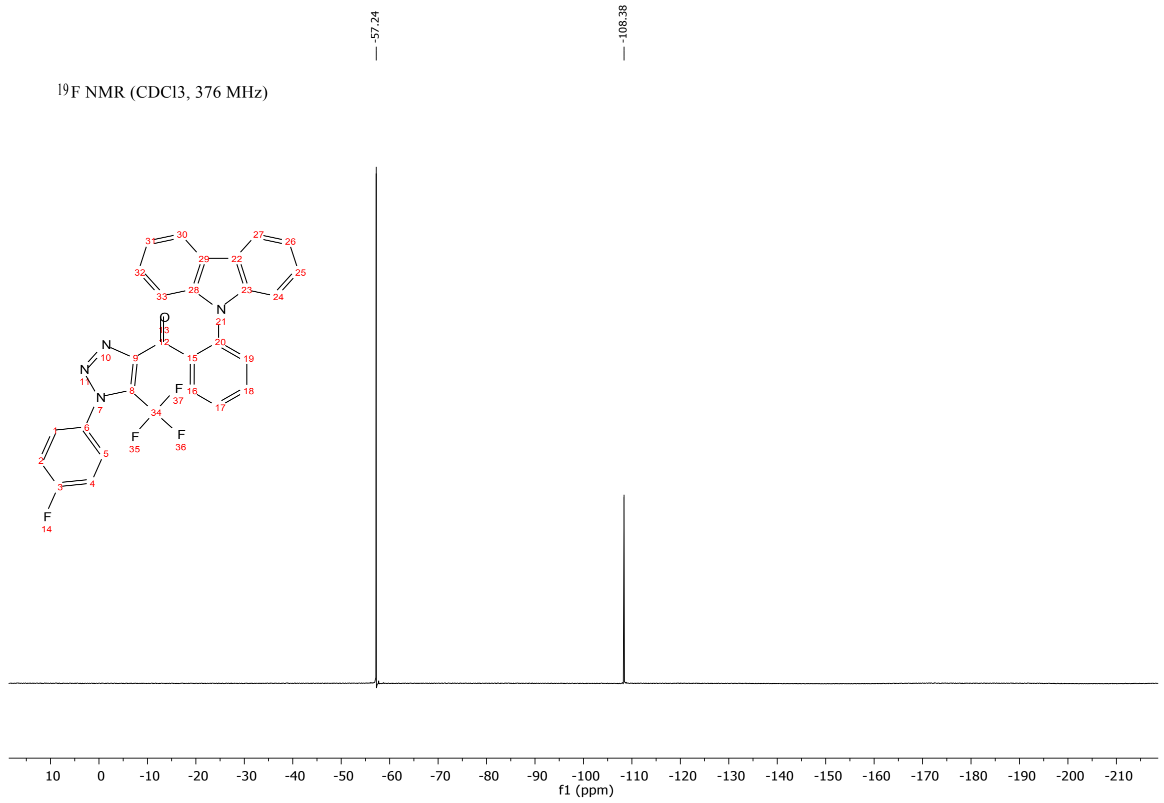

^{1}H NMR ($CDCl_3$, 400 MHz) (3-(9*H*-carbazol-9-yl)phenyl)(1-(4-fluorophenyl)-5-(trifluoromethyl)-1*H*-1,2,3-triazol-4-yl)methanone (5b):

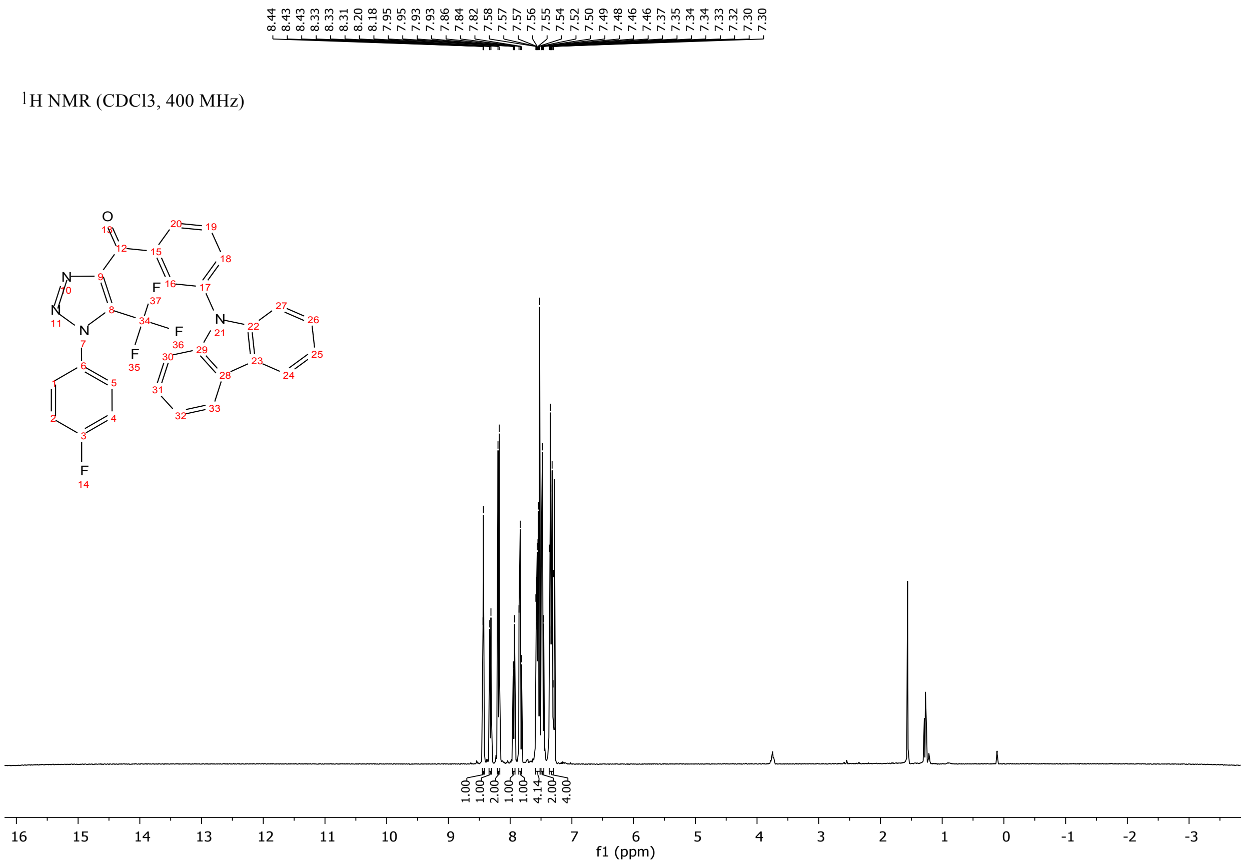

$^{13}C\{^{1}H\}$ NMR ($CDCl_3$, 126 MHz) (3-(9*H*-carbazol-9-yl)phenyl)(1-(4-fluorophenyl)-5-(trifluoromethyl)-1*H*-1,2,3-triazol-4-yl)methanone (5b):

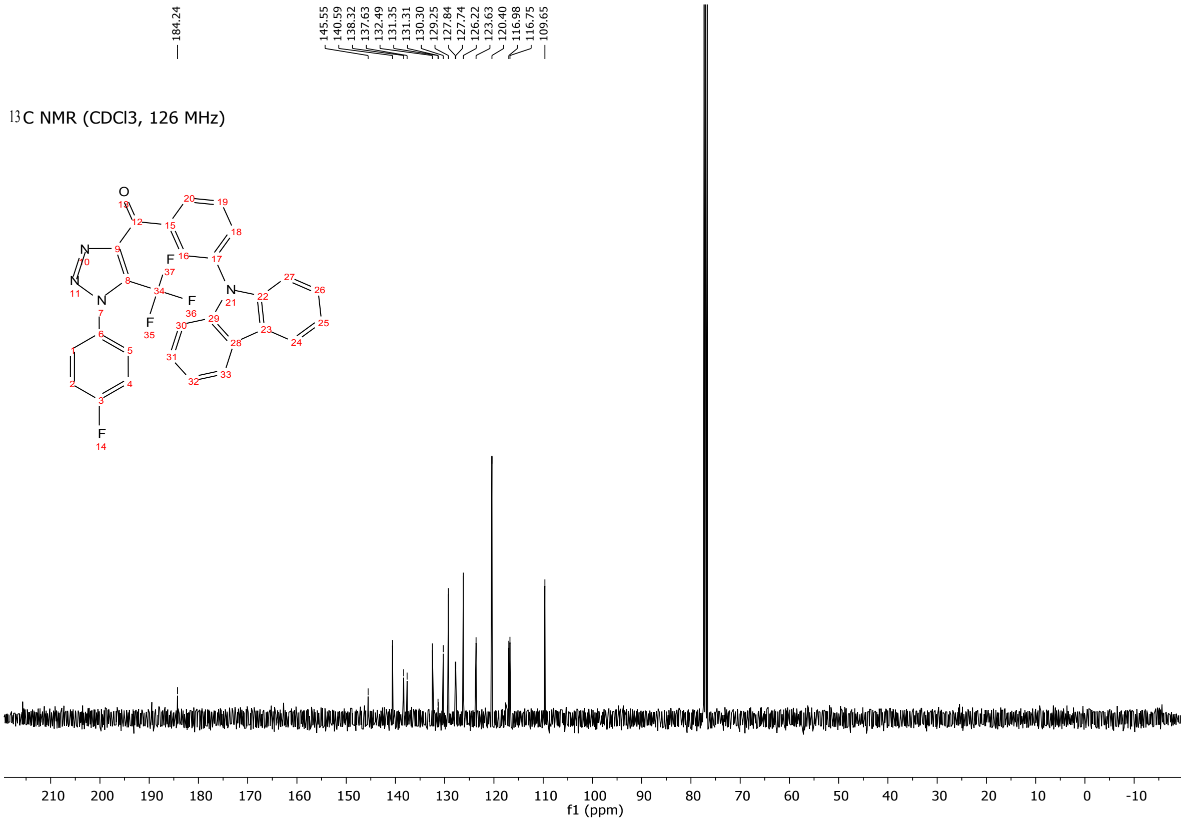

^{19}F NMR ($CDCl_3$, 376 MHz) (3-(9*H*-carbazol-9-yl)phenyl)(1-(4-fluorophenyl)-5-(trifluoromethyl)-1*H*-1,2,3-triazol-4-yl)methanone (5b):

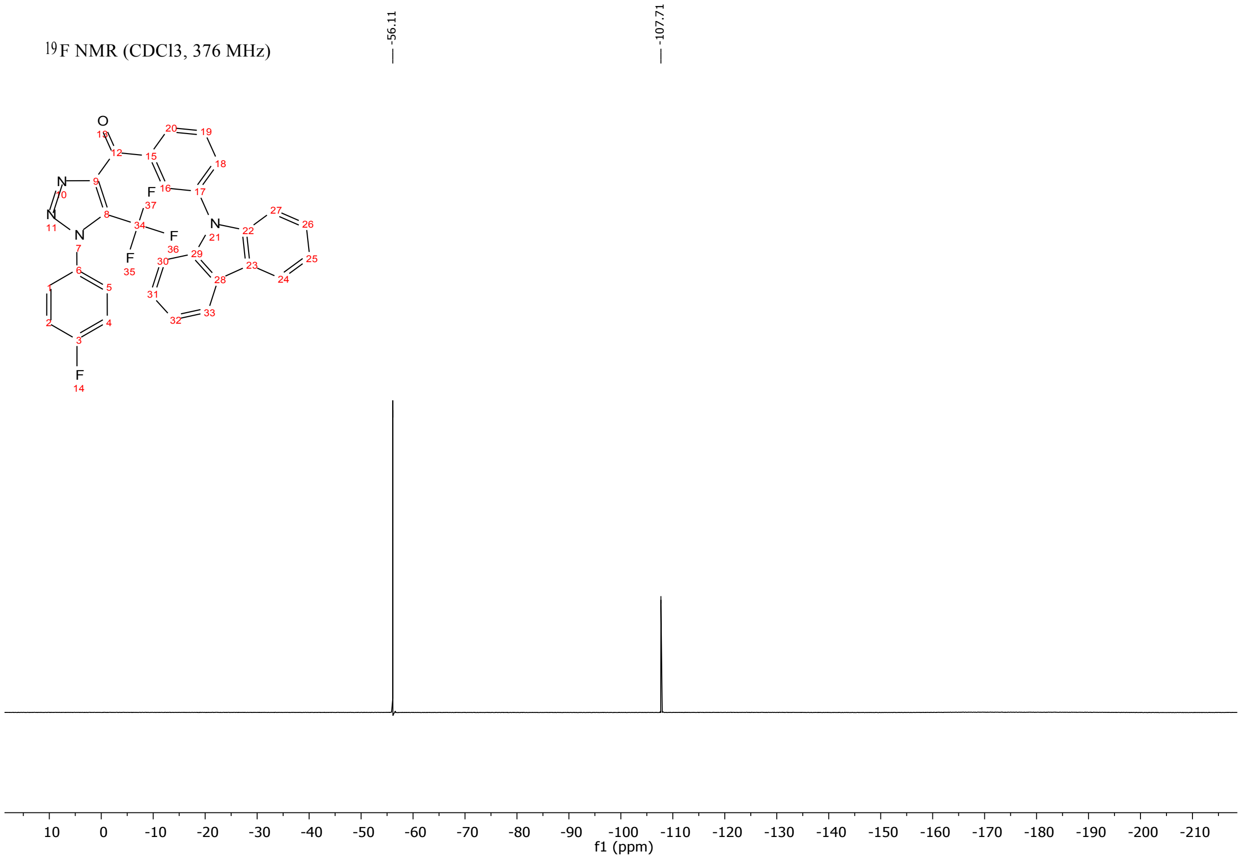

^{1}H NMR (DMSO-d_6, 400 MHz) (2,5-di(9*H*-carbazol-9-yl)phenyl)(1-(4-fluorophenyl)-5-(trifluoromethyl)-1*H*-1,2,3-triazol-4-yl)methanone (5c):

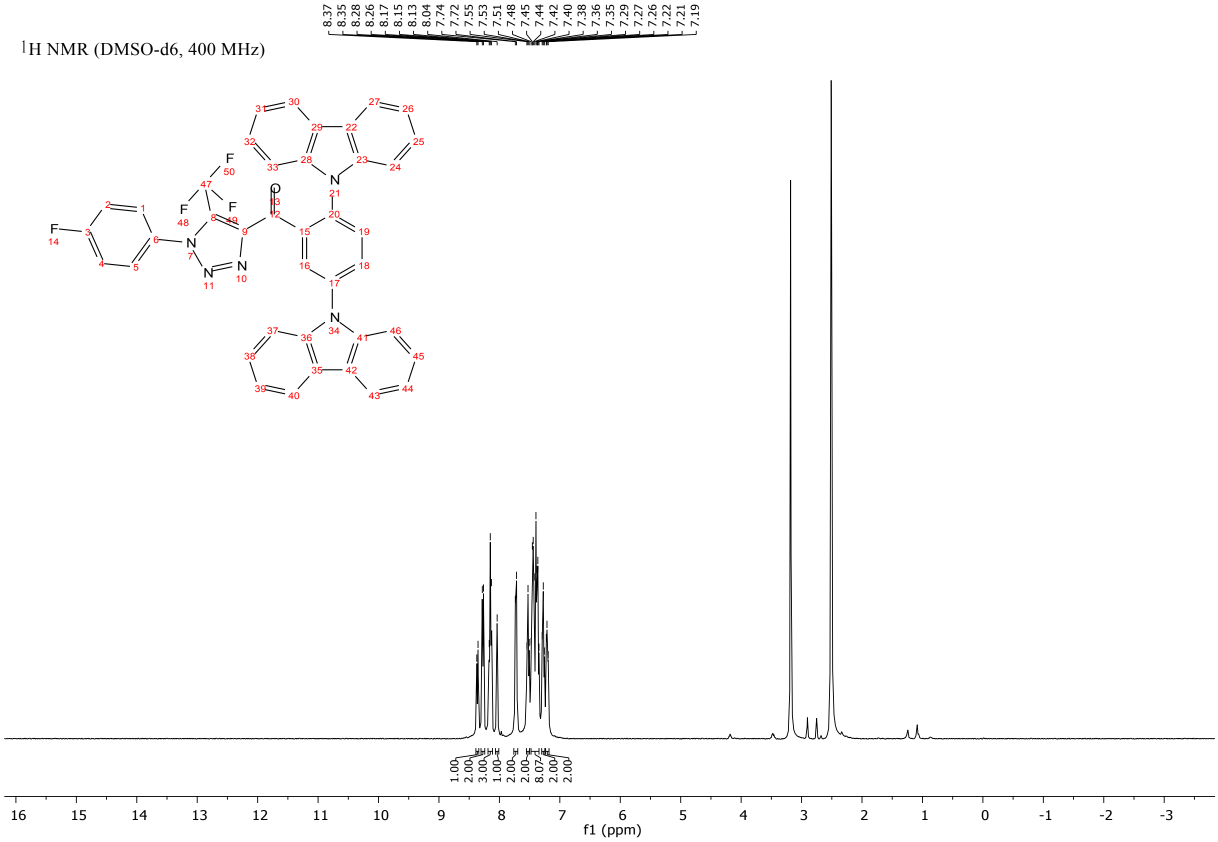

$^{13}C\{^{1}H\}$ NMR (DMSO-d_6, 126 MHz) (2,5-di(9*H*-carbazol-9-yl)phenyl)(1-(4-fluorophenyl)-5-(trifluoromethyl)-1*H*-1,2,3-triazol-4-yl)methanone (5c):

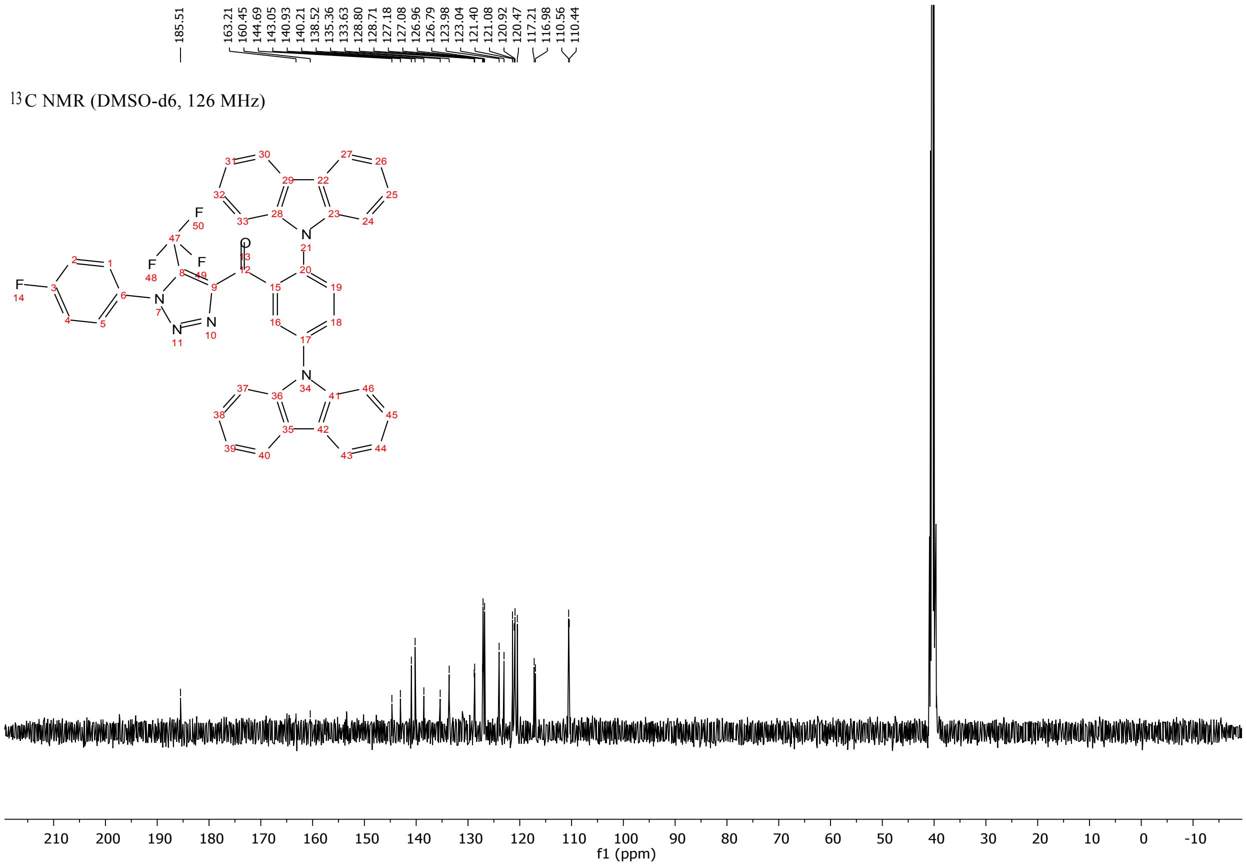

^{19}F NMR (DMSO-d_6, 376 MHz) (2,5-di(9*H*-carbazol-9-yl)phenyl)(1-(4-fluorophenyl)-5-(trifluoromethyl)-1*H*-1,2,3-triazol-4-yl)methanone (5c):

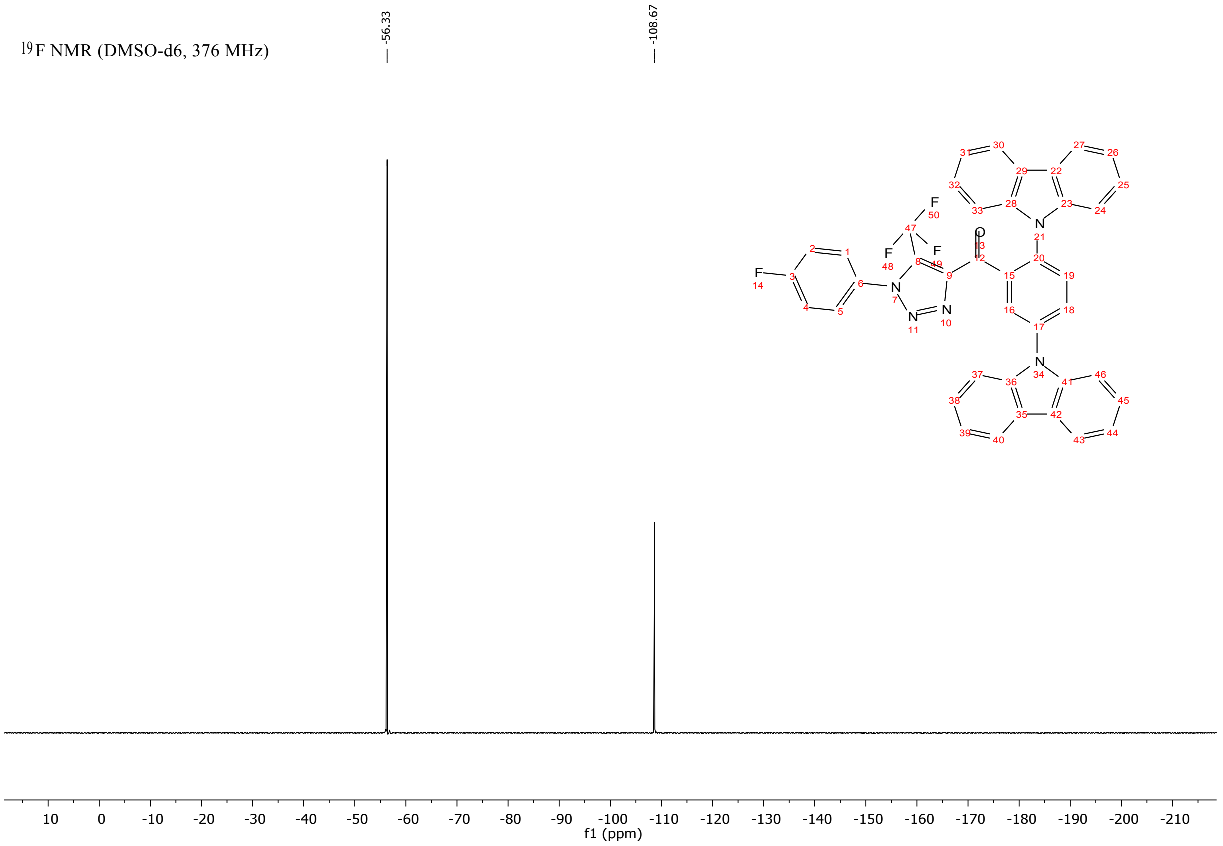

Table S1. Yields of triazole deriatives.

Entry		Bromine/Carbazole position to CO	Name	Overall yield
1	**5a**	*ortho*	**oCz4FAr3N** 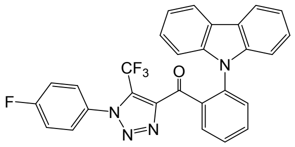	35%
3	**5b**	*meta*	**mCz4FAr3N** 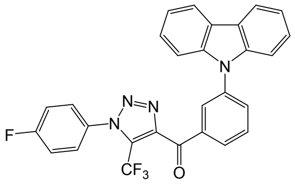	47%
13	**5c**	*ortho+meta*	**omCz4FAr3N** 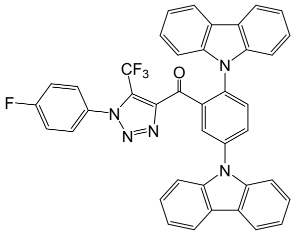	28%

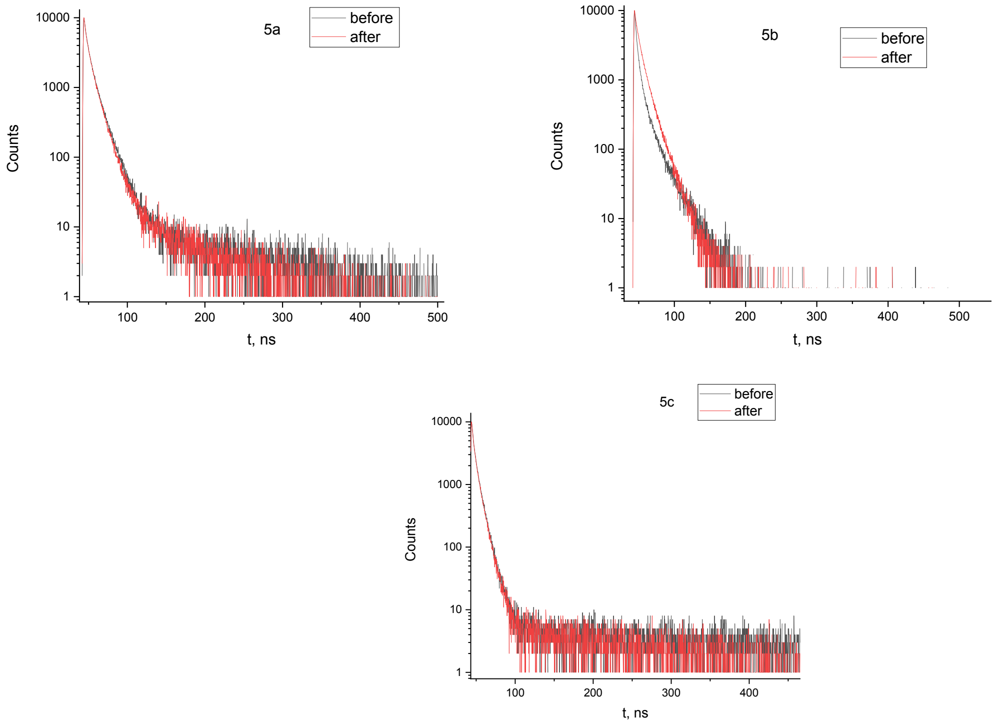

Figure S1. PL decay curves of the toluene solutions of compounds **5a-5c** recorded before and after deoxygenation.

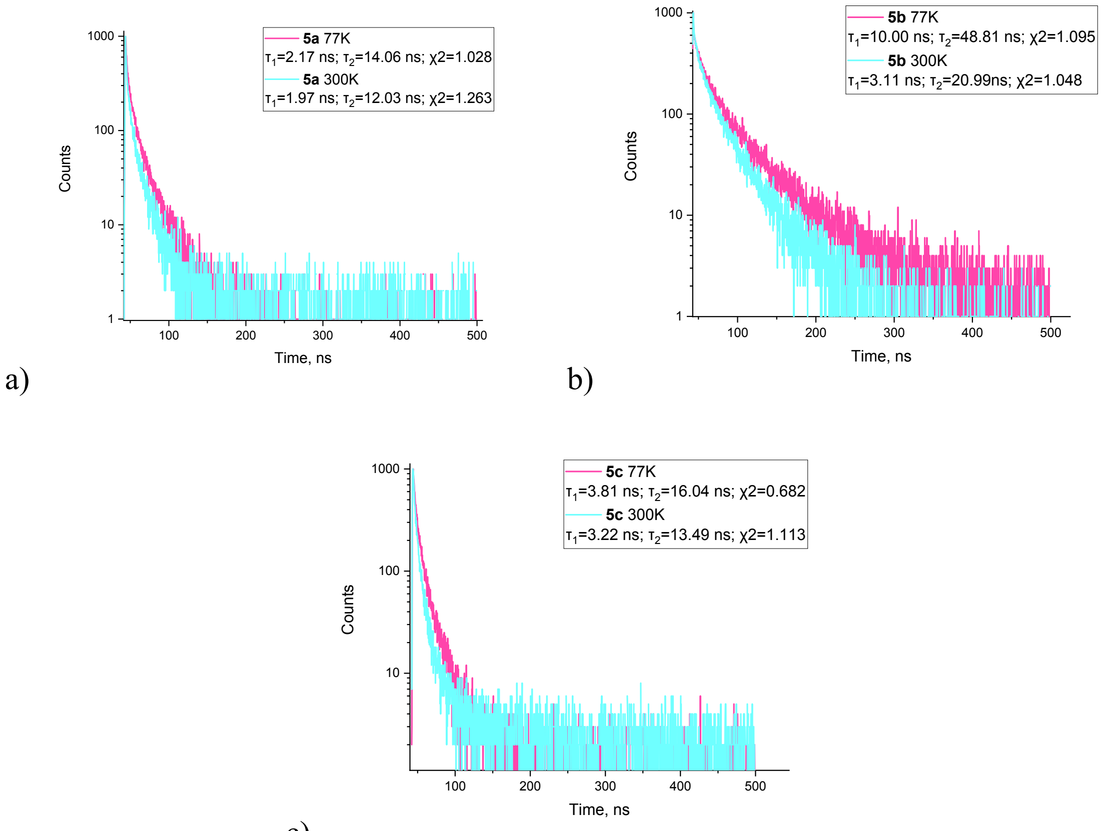

Figure S2. PL decay curves of the solid solutions of compounds **5a** (a), **5b** (c),**5c** (b) in Zeonex recorded at the different temperatures.

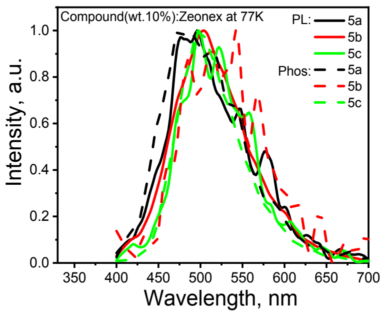

Figure S3. PL and phosphorescence (Phos.) of compound **5a**, **5b**, **5c** in Zeonex matrix recorded at 77K.

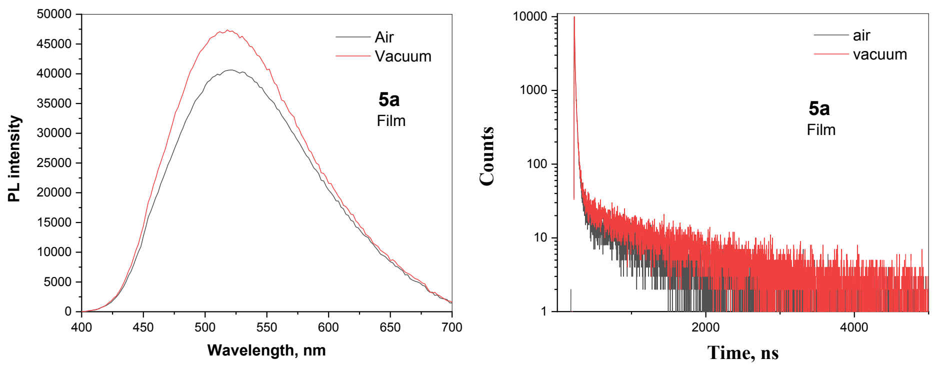

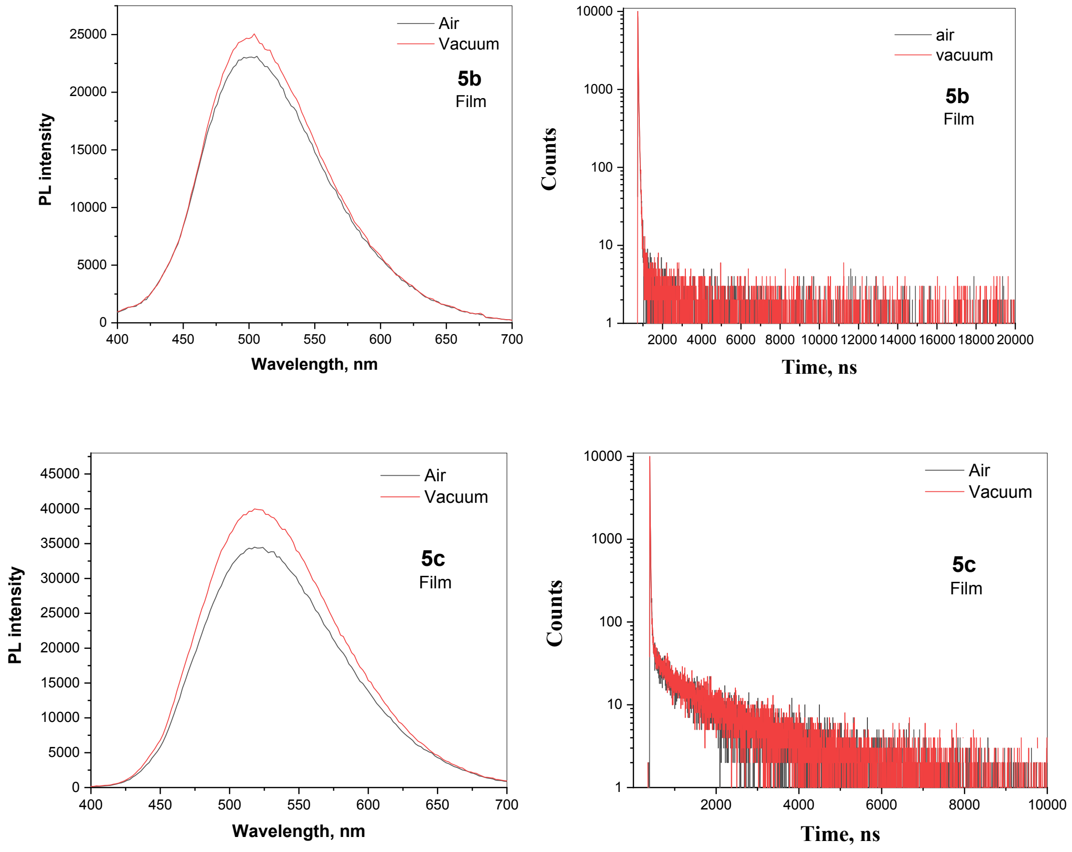

Figure S4. PL spectra (left) and PL decay curves (right) of the films of **5a-5c** in air and under vacuum.

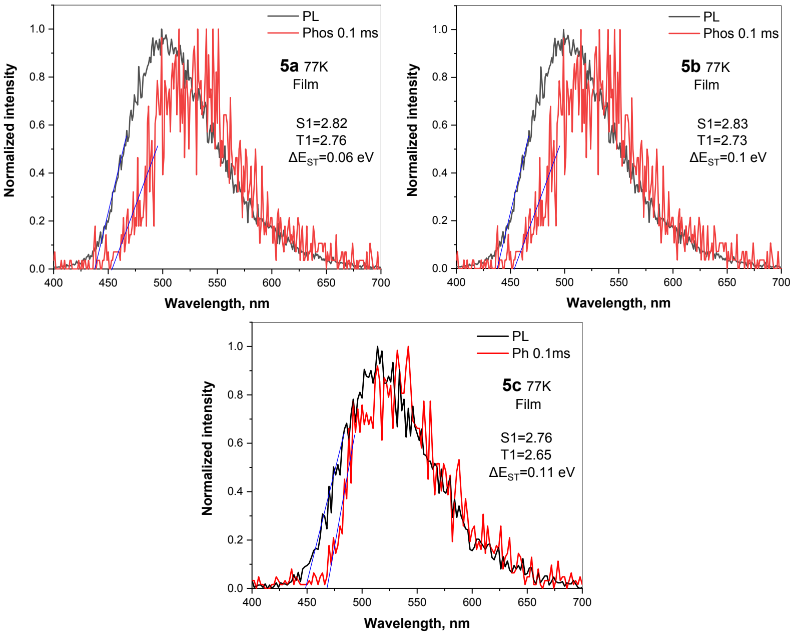

Figure S5. PL and phosphorescence spectra of the films of **5a-5c** recorded at 77K. Phosphorescence spectra were recorded using delay of 0.1 ms after excitation. Singlet S1 and triplet T1 levels were taken from the corresponding onsets as it is shown by the blue lines.

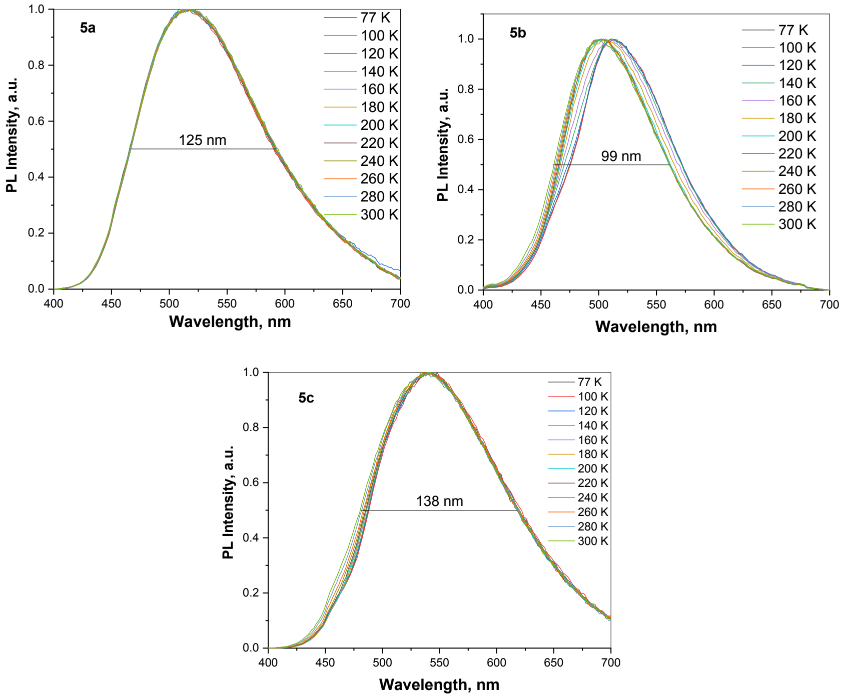

Figure S6. Normalized PL spectra of the films of **5a**, **5b** and **5c** recorded at the different temperatures.

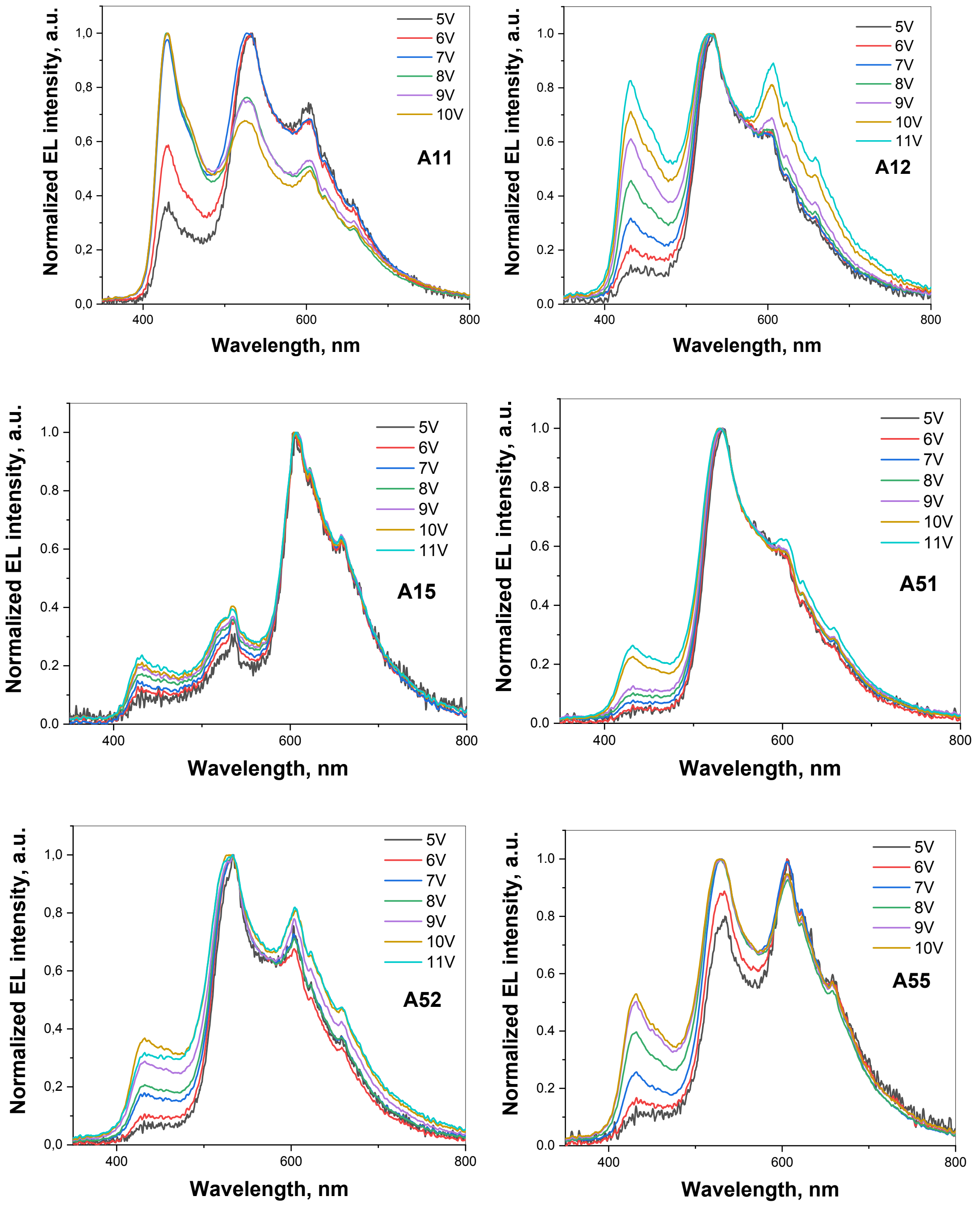

Figure S7. Normalized EL spectra of OLEDs recorded at the different voltages.

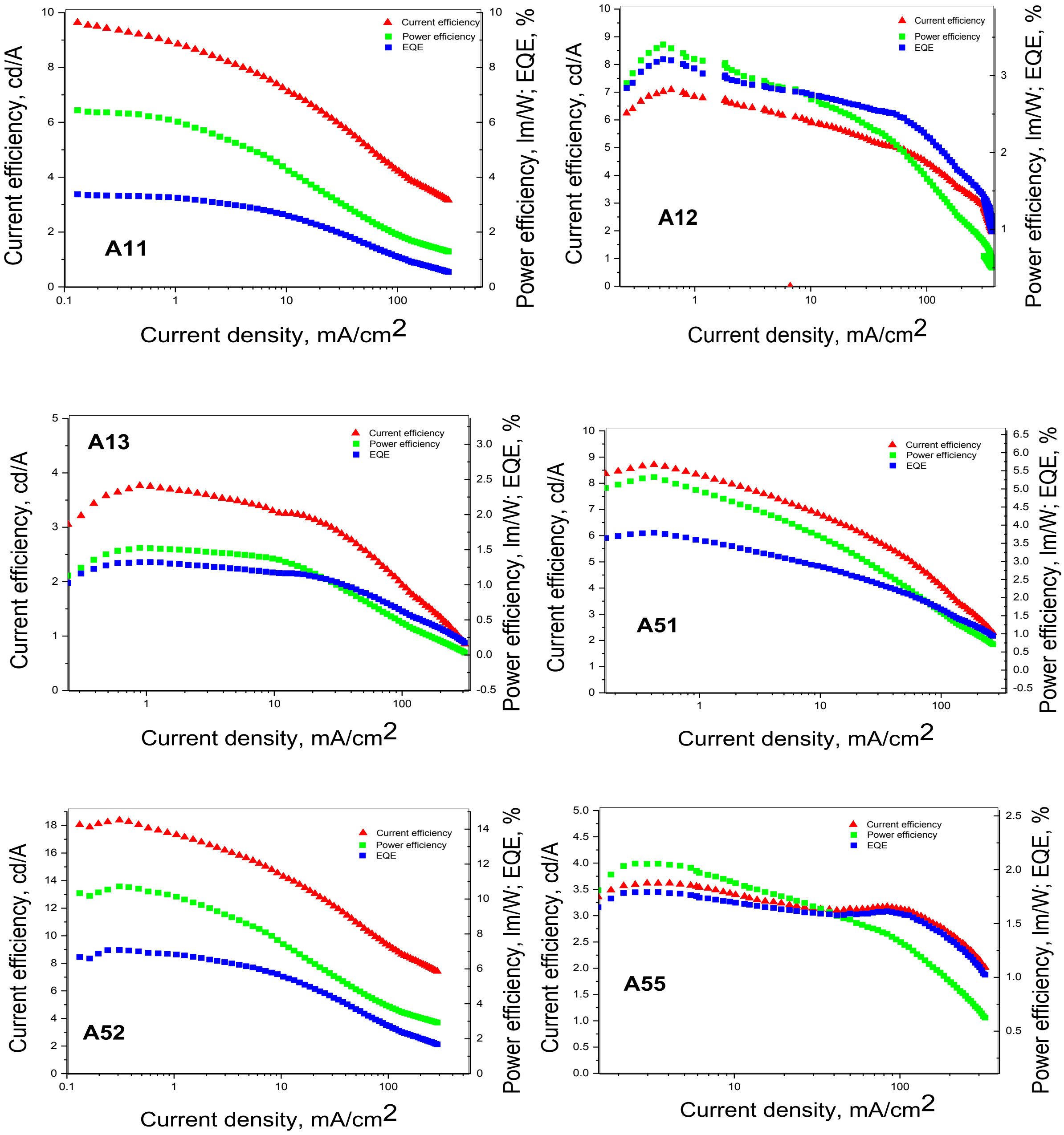

Figure S8. Current, power and external quantum efficiencies versus current density of OLEDs.

References

(1) Sambasiva Rao, P.; Kurumurthy, C.; Veeraswamy, B.; Santhosh Kumar, G.; Poornachandra, Y.; Ganesh Kumar, C.; Vasamsetti, S. B.; Kotamraju, S.; Narsaiah, B. Synthesis of Novel 1,2,3-Triazole Substituted-N-Alkyl/Aryl Nitrone Derivatives, Their Anti-Inflammatory and Anticancer Activity. *Eur. J. Med. Chem.* **2014**, *80*, 184–191. https://doi.org/10.1016/J.EJMECH.2014.04.052.

(2) Dai, Z.-C.; Chen, Y.-F.; Zhang, M.; Li, S.-K.; Yang, T.-T.; Shen, L.; Wang, J.-X.; Qian, S.-S.; Zhu, H.-L.; Ye, Y.-H. Synthesis and Antifungal Activity of 1,2,3-Triazole Phenylhydrazone Derivatives. *Org. Biomol. Chem.* **2014**, *13* (2), 477–486. https://doi.org/10.1039/C4OB01758G.

(3) Stevenson, R. J.; Azimi, I.; Flanagan, J. U.; Inserra, M.; Vetter, I.; Monteith, G. R.; Denny,

W. A. An SAR Study of Hydroxy-Trifluoromethylpyrazolines as Inhibitors of Orai1-Mediated Store Operated Ca2+ Entry in MDA-MB-231 Breast Cancer Cells Using a Convenient Fluorescence Imaging Plate Reader Assay. *Bioorg. Med. Chem.* **2018**, *26* (12), 3406–3413. https://doi.org/10.1016/J.BMC.2018.05.012.
(4) Büttner, S.; Riahi, A.; Hussain, I.; Yawer, M. A.; Lubbe, M.; Villinger, A.; Reinke, H.; Fischer, C.; Langer, P. First Synthesis of Functionalized 5-Aryl-3-(Trifluoromethyl)Phenols by Regioselective [3+3] Cyclocondensations of 1,3-Bis(Silyloxy)-1,3-Butadienes with 3-Aryl-3-Silyloxy-1-Trifluoromethyl-2-En-1-Ones. *Tetrahedron* **2009**, *65* (10), 2124–2135. https://doi.org/10.1016/j.tet.2008.12.076.
(5) Becke, A. D. Density-Functional Exchange-Energy Approximation with Correct Asymptotic Behavior. *Phys. Rev. A* **1988**, *38* (6), 3098–3100. https://doi.org/10.1103/PhysRevA.38.3098.
(6) Frisch, M. J.; Trucks, G. W.; Schlegel, H. B.; Scuseria, G. E.; Robb, M. A.; Cheeseman, J. R.; Scalmani, G.; Barone, V.; Petersson, G. A.; Nakatsuji, H.; Li, X.; Caricato, M.; Marenich, A.; Bloino, J.; Janesko, B. G.; Gomperts, R.; Mennucci, B.; Hratchian, H. P.; Ortiz, J. V.; Izmaylov, A. F.; Sonnenberg, J. L.; Williams-Young, D.; Ding, F.; Lipparini, F.; Egidi, F.; Goings, J.; Peng, B.; Petrone, A.; Henderson, T.; Ranasinghe, D.; Zakrzewski, V. G.; Gao, J.; Rega, N.; Zheng, G.; Liang, W.; Hada, M.; Ehara, M.; Toyota, K.; Fukuda, R.; Hasegawa, J.; Ishida, M.; Nakajima, T.; Honda, Y.; Kitao, O.; Nakai, H.; Vreven, T.; Throssell, K.; J. A. Montgomery, J.; Peralta, J. E.; Ogliaro, F.; Bearpark, M.; Heyd, J. J.; Brothers, E.; Kudin, K. N.; Staroverov, V. N.; Keith, T.; Kobayashi, R.; Normand, J.; Raghavachari, K.; Rendell, A.; Burant, J. C.; Iyengar, S. S.; Tomasi, J.; Cossi, M.; Millam, J. M.; Klene, M.; Adamo, C.; Cammi, R.; Ochterski, J. W.; Martin, R. L.; Morokuma, K.; Farkas, O.; Foresman, J. B.; Fox, D. J. Gaussian 09, Revision A.02. *Gaussian, Inc. Wallingford CT* **2016**.
(7) Gritzner, G.; Kuta, J. Recommendations on Reporting Electrode Potentials in Nonaqueous Solvents (Recommendations 1983). *Pure Appl. Chem.* **1984**, *56* (4), 461–466. https://doi.org/10.1351/pac198456040461.
